# The genome-wide impact of cadmium on microRNA and mRNA expression in contrasting Cd responsive wheat genotypes

**DOI:** 10.1186/s12864-019-5939-z

**Published:** 2019-07-29

**Authors:** Min Zhou, Shigang Zheng, Rong Liu, Lu Lu, Chihong Zhang, Lei Zhang, Levi Yant, Yu Wu

**Affiliations:** 1 Chengdu Institute of Biology, Chinese Academy of Sciences, Chengdu, 610041 Sichuan China; 2 University of Chinese Academy of Sciences, Beijing, 100049 China; 3 School of Life Sciences, University of Nottingham, Nottingham, NG7 2RD UK

**Keywords:** Wheat, RNA sequencing, microRNA, *HMA*

## Abstract

**Background:**

Heavy metal ATPases (HMAs) are responsible for Cd translocation and play a primary role in Cd detoxification in various plant species. However, the characteristics of HMAs and the regulatory mechanisms between HMAs and microRNAs in wheat (*Triticum aestivum* L) remain unknown.

**Results:**

By comparative microRNA and transcriptome analysis, a total three known and 19 novel differentially expressed microRNAs (DEMs) and 1561 differentially expressed genes (DEGs) were found in L17 after Cd treatment. In H17, by contrast, 12 known and 57 novel DEMs, and only 297 Cd-induced DEGs were found. Functional enrichments of DEMs and DEGs indicate how genotype-specific biological processes responded to Cd stress. Processes found to be involved in microRNAs-associated Cd response include: ubiquitin mediated proteolysis, tyrosine metabolism, and carbon fixation pathways and thiamine metabolism. For the mRNA response, categories including terpenoid backbone biosynthesis and phenylalanine metabolism, and photosynthesis - antenna proteins and ABC transporters were enriched. Moreover, we identified 32 TaHMA genes in wheat. Phylogenetic trees, chromosomal locations, conserved motifs and expression levels in different tissues and roots under Cd stress are presented. Finally, we infer a microRNA-TaHMAs expression network, indicating that miRNAs can regulate TaHMAs.

**Conclusion:**

Our findings suggest that microRNAs play important role in wheat under Cd stress through regulation of targets such as TaHMA2;1. Identification of these targets will be useful for screening and breeding low-Cd accumulation wheat lines.

**Electronic supplementary material:**

The online version of this article (10.1186/s12864-019-5939-z) contains supplementary material, which is available to authorized users.

## Background

Cadmium (Cd) contamination of soils is rapidly increasing from both human activities and other environmental causes [1]. Deleterious effects of Cd are dramatic and varied in both humans and plants. In humans, Cd ingestion leads to osteoporosis and emphysema, among other diseases [2, 3]. In plants, high Cd results in a wide range of biochemical and physiological disorders, inhibiting yield and quality [4]. Unfortunately, even moderate levels of Cd in soils can result in dramatic Cd accumulation in leaves or grains [5, 6]. Contamination in the food chain with Cd thus poses a major widespread hazard to human health [7–9]. Wheat, one of the most important worldwide crops, is grown on nearly 20% of cultivated land and serves as a staple food source for 30% of humanity [10, 11]. Previous studies indicated that compared with other cereals, wheat accumulates Cd primarily via roots, eventually accumulating Cd in edible grains [12, 13]. Therefore, it is very important to understand wheat responses to Cd in order to improve wheat growth, grain quality, and human welfare.

MicroRNAs (miRNAs) are typically 21 nucleotide endogenous non-coding RNAs that play key roles in regulating gene expression post-transcriptionally, usually via cleavage or translational repression of target mRNAs [14, 15]. In plants, miRNAs are involved in various physiological processes and play important roles in growth and development [16, 17], and numerous miRNAs participate in responses to biotic and abiotic stresses [18–21]. Deep-sequencing of developing wheat grains revealed 605 miRNAs [22] and 57 conserved miRNA families have been identified in wheat hybrid necrosis [23]. Hundreds of miRNAs were differentially expressed in leaf and roots tissues in wheat under drought stress, respectively [24]. Individual analysis of miRNAs or mRNAs have been widely reported under various stressors. However, the interplay of Cd, regulatory miRNAs, and the transcriptome is not at all understood, so there is still an obvious gap in our understanding of their interplay in a major crop.

P_1B_-ATPases, also known as heavy metal ATPases (HMAs), are proteins that use ATP to pump a wide range of cations across membranes against electrochemical gradients [25]. AtHMA2 and AtHMA4 act as pumps to efflux Zn/Cd out of cells, playing critical role in root-shoot Zn/Cd translocation through xylem loading [26]. The HMA3 proteins, such as AtHMA3, TcHMA3 and OsHMA3, are typically located in tonoplasts and are not regulated through Zn or Cd; these proteins play a role in pumping Zn and/or Cd into vacuoles [27–29]. Much available evidence indicates that HMAs play an important role in heavy metal transmembrane transport. However, there is little understanding of HMAs in the wheat genome and their roles in wheat under Cd stress.

Therefore, to gain an understanding of the global transcriptional response and its interplay with miRNAs and HMAs in wheat during Cd stress, we used next generation transcriptome and miRNA sequencing to profile the responses of low- and high- cadmium-accumulating wheat cultivars in response to Cd. Additionally, we paid special attention to regulatory dynamics of the HMA gene family. We further verified by qRT-PCR significantly differentially expressed of miRNAs, mRNAs and TaHMAs. Finally, we performed for gene ontology and pathway analysis, as well as depicted general functional landscapes of miRNAs-TaHMAs expression. We expect that these findings will provide a meaningful resource for the understanding the global transcriptional response to Cd and how these are modulated by miRNAs.

## Results and discussion

### Differences in cd accumulation in grains among wheat germplasms

**Fig. 1:**
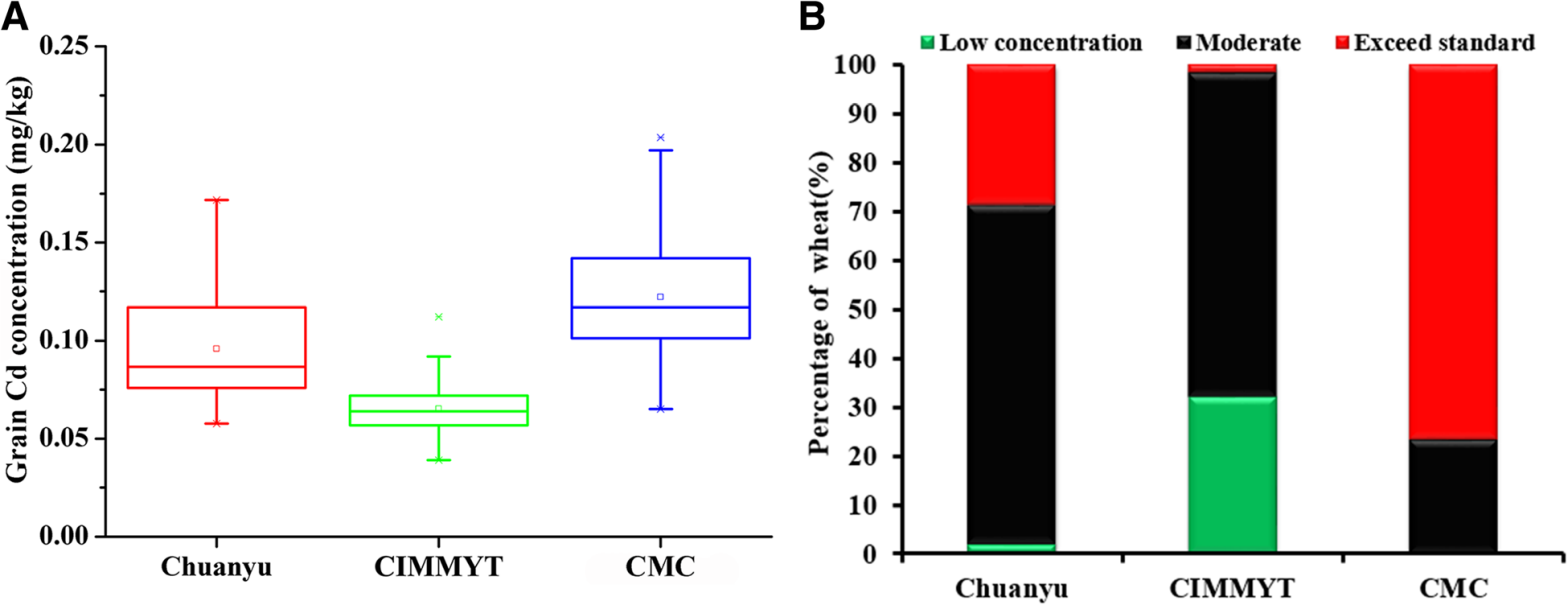
**a** Grain Cd concentration among three major wheat groups. The Chuanyu group included 52 wheat cultivars, the CIMMYT group included 56 wheat cultivars, and the CMC group included 73 wheat cultivars. **b** Percentage of low Cd concentration (≤ 0.0579 mg/kg), moderate Cd concentrations (< 0.1 mg/kg) and exceeding Cd concentration standard (≥ 0.1 mg/kg) wheat in each of the three groups

We first screened 185 wheat germplasms for grain Cd levels using ICP-MS (Additional file 1: Table S1). By group, the average concentrations of Cd in grain decreased in the order of CMC > Chuanyu > CIMMYT, with significant differences between group averages (Fig. 1a). The tolerance limit of Cd in food is 0.1 mg/kg set by Ministry of Health of PRC (People’s Republic of China). Thus, there were 15 (28.85%) cultivars that exceeded the tolerance limit in Chuanyu group, 1 (1.79%) in the CIMMYT group, and 56 (76.71%) in the CMC group (Fig. 1b). Our results revealed that the average grain Cd concentration of four durum wheat genotypes was 0.224 mg/kg, and ranged from 0.191 to 0.371 mg/kg. These four durum cultivars contained higher grain Cd concentrations compared with bread wheat (Additional file 2: Figure S1), in agreement with previous studies [30–32]. Previous work showed that durum wheat cultivars grown in a variety test in South Dakota contained similar average grain Cd concentrations (0.22 mg/kg, ranged from 0.13 to 0.25 mg/kg) [33], indicating that our results are good representatives of world-wide sampling.

### Differentially expressed miRNAs and mRNAs between L17Cd and L17CK, H17Cd and H17CK

To illuminate the underlying molecular mechanism, we chose cultivars strongly contrasting for Cd content: a low-Cd-accumulating cultivar (Chuanyu 17, hereafter L17) and a high-Cd-accumulating cultivar (a Chuanyu 17 sib-line, hereafter H17) to determine differentially expressed miRNAs and mRNAs in roots. We generated 12 small RNA sequencing libraries from three biological replicates each for L17 and H17 roots under control and CdCl_2_ treatment and sequenced them on an Illumina HiSeq TM 2500. The major characteristics of these libraries are summarized in Table 1. Between 18.8 and 32.4 million total raw reads per library were obtained. After quality filtration, between 12.6 and 20.4 million clean reads were obtained per replicate.

**Table 1:** Read statistics in small RNA libraries by genotype

Type	L17CK	L17Cd	H17CK	H17Cd
Raw reads	29,634,338 ± 12,235,540	23,765,742 ± 5,243,896	18,786,733 ± 2,145,550	32,365,435 ± 7,244,882
Clean reads	20,430,195 ± 8,138,193	14,922,483 ± 7,059,238	12,556,082 ± 1,740,984	19,285,729 ± 5,444,137
Unique clean reads	3,232,322 ± 981,148	2,426,636 ± 1,239,154	2,313,643 ± 314,127	2,580,979 ± 647,449
Wheat genome-matched read (redundant)	17,858,724 ± 7,263,840	12,629,611 ± 5,018,126	8,991,791 ± 1,289,534	14,299,057 ± 3,343,128
Wheat genome-matched read (unique)	2,180,300 ± 664,560	1,604,078 ± 683,985	1,313,826 ± 142,562	1,481,392 ± 264,618
Wheat genome-matched read (redundant %)	87.14 ± 1.14	86.43 ± 5.95	71.61 ± 2.78	74.81 ± 3.80
Wheat genome-matched read (unique %)	67.45 ± 0.56	67.82 ± 5.18	56.93 ± 1.82	58.12 ± 4.71

In each wheat samples, data is shown as the mean of the three libraries generated ± standard deviation

After comparing the distributions of miRNA expression values in the samples after normalization and correlation analysis of miRNA expression levels between samples and samples (Additional file 2: Figure S2), we next performed miRNA expression analysis and found many clear contrasts in miRNA expression between genotypes and groups. Two miRNAs were upregulated in L17Cd (treated with 100 μM CdCl_2_ for 24 h Cd) and one was downregulated compared with L17CK (CdCl_2_-free). In the high-Cd-accumulating genotype (H17), 12 miRNAs in H17Cd (treated with 100 μM CdCl_2_ for 24 h Cd) were downregulated compared with H17CK (CdCl_2_-free) (Table 2). Regarding novel miRNAs in L17, 10 miRNAs were upregulated and nine were downregulated in L17Cd compared with L17CK, while in H17, 49 miRNAs were upregulated and eight were downregulated in H17Cd compared with H17CK (Table 3). In addition, one uniquely expressed miRNA between L17Cd and L17CK, one uniquely expressed miRNA between H17Cd and H17CK, and two expressed miRNAs were commonly expressed. These results indicated that tae-miR9664-3p and tae-miR159a were upregulated in L17Cd group compared with that in L17CK group, while they were downregulated in H17Cd group compared with that in H17CK group. Moreover, according to our RNA sequencing data, the putative target mRNAs (such as Traes_7BL_66C9AAA60, Traes_1BS_1E568BC28, Traes_5DL_BDE25A669, Traes_2DS_47DDC83DC and Traes_5BS_C3B48D8CC) for upregulated tae-miR9664-3p and the putative target mRNAs (such as Traes_3AL_F09C7CA19, Traes_7AS_2831C6473, Traes_7DS_0FFF2FB02 and Traes_4AL_03723C140) for upregulated tae-miR159a were downregulated in L17Cd group compared with that in L17CK group. Previous work demonstrated that tae-miR-9664 was expressed in the flag leaf and developing seed of wheat (*Triticum aestivum* L.) [34]. Tae-miR159a played positive roles in wheat response to *Puccinia striiformis* f.sp.*tritici* through the regulation of *TaMyb3* expression [35]. Our results suggested that contrasting Cd-accumulating ability of L17 and H17 might associated with the up-regulation or down-regulation between tae-miR9663-3p, tae-miR159a and their target genes. We also found that tae-miR9774 was uniquely down-regulated between L17Cd and L17CK, while tae-miR398 was specifically down-regulated between H17Cd and H17CK. A previous study demonstrated that tae-miR-9774 was expressed in wheat leaves, stems, roots and young spikes [36]. miR398 was induced by UVB light and heat stress in Arabidopsis but was inhibited by ABA, salinity, cold, and oxidative stress [37, 38]. miR398 can regulate copper superoxide dismutase (CSD) 1 and 2 that were both induced by salinity treatment [37, 39]. These results suggested that miRNAs could participate in the growth and development of wheat and also function in wheat response to both biotic and abiotic stress. To our knowledge, this is the first study to identify differentially expressed miRNAs in response to Cd accumulation in multiple wheat genotypes contrasting for Cd accumulation.

**Table 2:** Known miRNAs identified in the 12 wheat sRNA libraries

Comparison	miRNA family	miRNA_id	log2FoldChange	P	Down
L17Cd/L17CK	miR159a	tae-miR159a	0.87	3.347E-05	Up
	miR9664	tae-miR9664-3p	0.53	0.0427885	Up
	miR9774	tae-miR9774	−2.29	0.0279111	Down
H17Cd/H17CK	miR159a	tae-miR159a	−1.04	5.42E-11	Down
	miR167	tae-miR167a	−1.47	0.0379267	Down
	mi395	tae-miR395b	−2.40	0.0038538	Down
	miR397	tae-miR397-5p	−1.50	0.019139	Down
	miR398	tae-miR398	−0.99	0.0250389	Down
	miR5048	tae-miR5048-5p	−0.91	0.0247939	Down
	miR7757	tae-miR7757-5p	−1.37	0.0001656	Down
	miR9662	tae-miR9662a-3p	−1.10	7.92E-06	Down
	miR9664	tae-miR9664-3p	−0.68	0.0203362	Down
	miR9669	tae-miR9669-5p	−1.50	0.0258714	Down
	miR9674	tae-miR9674b-5p	−0.92	0.0109898	Down
	miR9778	tae-miR9778	−1.30	1.11E-07	Down

**Table 3:** Summary of newly identified novel miRNAs in the 12 wheat sRNA libraries

Comparison	miRNA_id	log2 (Fold Change)	P	Down	Mature sequence(5'-3')	Length
L17Cd/L17CK	2B_20696^a^	1.69	0.0270291	Up	CUCGCCGGUCGCGCGUCCUCC	21
	2B_21940^a^	2.33	0.0462198	Up	AUGACACGGGGAGGUAGC	18
	2B_39534^a^	6.11	0.0176417	Up	AGGCUGGAGGAACGUAGG	18
	2B_40590^a^	2.00	0.0383352	Up	GUGUGGAGCAAAAGGGUG	18
	4B_10717^a^	4.18	0.0021475	Up	CCCCGGGUGCGAGCUCCU	18
	4B_12465^a^	5.45	0.0221443	Up	CCUCCUGGUGAGCCUGCG	18
	4B_4791^a^	1.96	0.0091409	Up	UGGGGUUGUGGCAAUGGCC	19
	4B_519^a^	2.40	0.0050086	Up	AAUCUGGUUGUCGCCUCC	18
	4B_5711^a^	4.20	0.0024414	Up	GUGAAGAGACAUGGAGGU	18
	4B_9306^a^	1.13	0.0050015	Up	UAGAAUGGCUGGUGCUAUGGA	21
	2B_19887^a^	-4.18	0.0352225	Down	ACGGACCGCGCUACUACUAUAAGU	24
	2B_22021^a^	-1.39	0.016954	Down	AUGGAAGACGUGGUAGCC	18
	2B_28100^a^	-1.27	0.0412731	Down	AGGUAGAGAGGAAGGUGGG	19
	2B_33543^a^	-3.40	0.0480069	Down	CUCGCCGGAGGAGCGUGC	18
	2B_38598^a^	-2.15	0.0214449	Down	CUGCUGGUGCUGUAGCCCU	19
	4B_17875	-4.36	0.0138807	Down	UUCCAAUUUACUCGUCGUGGU	21
	4B_4344^a^	-2.68	0.0327648	Down	GGUGUGGACCAGGUUUCC	18
	4B_87^a^	-4.35	0.0106819	Down	CGCGUGCAGGAUGAAGCC	18
	4B_8797	-2.08	0.0458935	Down	ACUCACUCUGUAAACUAAUAUAAG	24
H17Cd/H17CK	2B_19349^a^	4.00	1.83E-06	Up	AAACAGUCUGUAAAGCCCC	19
	2B_20999^a^	2.42	1.06E-14	Up	CUACGGGGGAAAGCAGGG	18
	2B_21614^a^	2.02	0.0023063	Up	UGUUGAAGUAGCUGGAAC	18
	2B_21995^a^	1.41	0.002425	Up	CGGAGAGGGGAGUGAAGU	18
	2B_22021^a^	2.93	9.43E-27	Up	AUGGAAGACGUGGUAGCC	18
	2B_22879^a^	3.19	0.0120242	Up	CGGCCAUCGUGAGGUUAGUGC	21
	2B_25173^a^	2.36	0.0000977	Up	GACUUUGAUCCAGAGAUCA	19
	2B_27180^a^	1.86	0.0252563	Up	GUUGGUCGGGAGUUCGAUCCU	21
	2B_28100^a^	1.76	4.01E-07	Up	AGGUAGAGAGGAAGGUGGG	19
	2B_28714^a^	1.46	0.0001773	Up	AUGAGAGGACGGACAAGG	18
	2B_29589^a^	1.98	0.0040311	Up	GAGUUUGGGAGUCUGUGUGU	20
	2B_32148	1.66	0.0364154	Up	UCAGUGCAAUCCCUCUGGAAU	21
	2B_33858^a^	1.58	0.0226808	Up	CGUGCCAGCAGCCGCGGA	18
	2B_37122^a^	1.33	0.0002491	Up	UCUGGUGGAUGCCUUGGG	18
	2B_37173^a^	1.15	0.0031618	Up	AGAGGGUGGUGGAGUUUCU	19
	2B_38197^a^	5.03	1.57E-10	Up	UUGUUGGAUAUGUUGGUU	18
	2B_40139^a^	2.06	0.0018813	Up	GUGGAUGCCUUGGCGAUC	18
	2B_40352^a^	2.74	0.0074044	Up	CAAUGGUAGAGCAGAGGC	18
	2B_42257^a^	1.93	0.0031222	Up	GGCGGCGGACGGGUGAGC	18
	2B_42905^a^	0.91	0.0142497	Up	GAGGUGCUGCAUGGCUGG	18
	2B_43522^a^	1.91	0.0003365	Up	UGAGGUUUGAGAGGGGCU	18
	4B_10203^a^	1.29	0.0034214	Up	GAGGCGAUGAAGGACGGG	18
	4B_10621^a^	1.88	0.0391309	Up	GGGUGAAGCUGUGGCGCG	18
	4B_10870^a^	2.94	6.11E-06	Up	UGGUUCUGUAUGGAAGUG	18
	4B_12067^a^	1.14	0.0151284	Up	AUGAACGCUAGCGGGAGG	18
	4B_12312^a^	3.38	0.0022606	Up	GAGUGGUCGAGCGGGACGG	19
	4B_13500^a^	2.41	0.0044361	Up	GGUUCUGUAUGGAAGUGG	18
	4B_13640^a^	1.37	0.0041213	Up	AGGUCUGGGUUCGAGUCC	18
	4B_1373^a^	2.42	0.0134111	Up	GCUUGUCGCUGGUUUGAG	18
	4B_15373^a^	0.92	0.0420051	Up	GUGGCUCCACGUGGGCGGGC	20
	4B_15496^a^	1.63	0.0036066	Up	UGCGGGAUGGAGCAGUCG	18
	4B_15927^a^	2.37	2.51E-08	Up	AUGCUAGUCGAGCGGAUA	18
	4B_16269^a^	3.22	3.8E-11	Up	AGGUCGACGGUUCGAAUCC	19
	4B_16562^a^	1.34	0.0445484	Up	CGGAGGUCGCGGGUUCGA	18
	4B_16625^a^	1.94	0.0000161	Up	UCCUGGGGUUGGAGAAGG	18
	4B_18394^a^	1.24	0.007484	Up	GCGGCGAGAGCGGGUCGCU	19
	4B_1943^a^	1.85	0.0000179	Up	ACAGUGGAUGCCUUGGCA	18
	4B_2104^a^	1.61	0.0136994	Up	AAGCCUCUGGCGAAUGGG	18
	4B_2324^a^	3.28	0.0050367	Up	GAUUUAUCGGGGAAGGAU	18
	4B_2847^a^	3.91	0.0001901	Up	GAGGAAGGUGGAUGACGG	18
	4B_4039^a^	3.73	0.0200414	Up	GAACGGAAGUUGGGGGCG	18
	4B_4609^a^	3.46	0.0000312	Up	GAUCUUGAUGUGGUGUGCA	19
	4B_4703^a^	2.74	1.23E-07	Up	GUGAUUCAGCGGCGGACG	18
	4B_5897^a^	1.35	0.0138282	Up	AGGAGGGUGGGGAUGAGGU	19
	4B_5947^a^	2.75	0.0124835	Up	GAGUGGUCGAGCGGGACGG	19
	4B_6530^a^	2.05	3.8E-10	Up	GCUCGUGUCGUGAGCUGG	18
	4B_841^a^	1.64	0.0087137	Up	GAAGGUCGUGGGUUCGUG	18
	4B_88^a^	3.20	0.0471916	Up	AGACGUUUCCGUCGACUACGAAA	23
	4B_974^a^	1.79	0.0009387	Up	UAUCGUGGGUUCGAAUCC	18
	2B_19630^a^	-1.52	0.0134799	Down	CAGCCUGCCAACCCUGGG	18
	2B_23520^a^	-5.38	3.24E-07	Down	GCUUCGGCGGUAUCCUCAU	19
	2B_27285^a^	-1.72	0.0294342	Down	GAUGGGUCUCUGAGGGAU	18
	2B_40624^a^	-1.93	0.0212515	Down	UGGCGAUGAGGAACGGAA	18
	2B_40888^a^	-2.07	0.0151122	Down	GGCGGAGGCUGCGGGUUU	18
	4B_11590^a^	-2.33	0.0093351	Down	GAGGGGUGUUGAUUUUCCGG	20
	4B_7298^a^	-1.77	0.0132316	Down	GAGGGGUGUUGAUUUUCCGG	20
	4B_7309^a^	-2.87	0.0008129	Down	GAACGGAAGUUGGGGGCG	18

^a^Highly conserved miRNAs

**Fig. 2:**
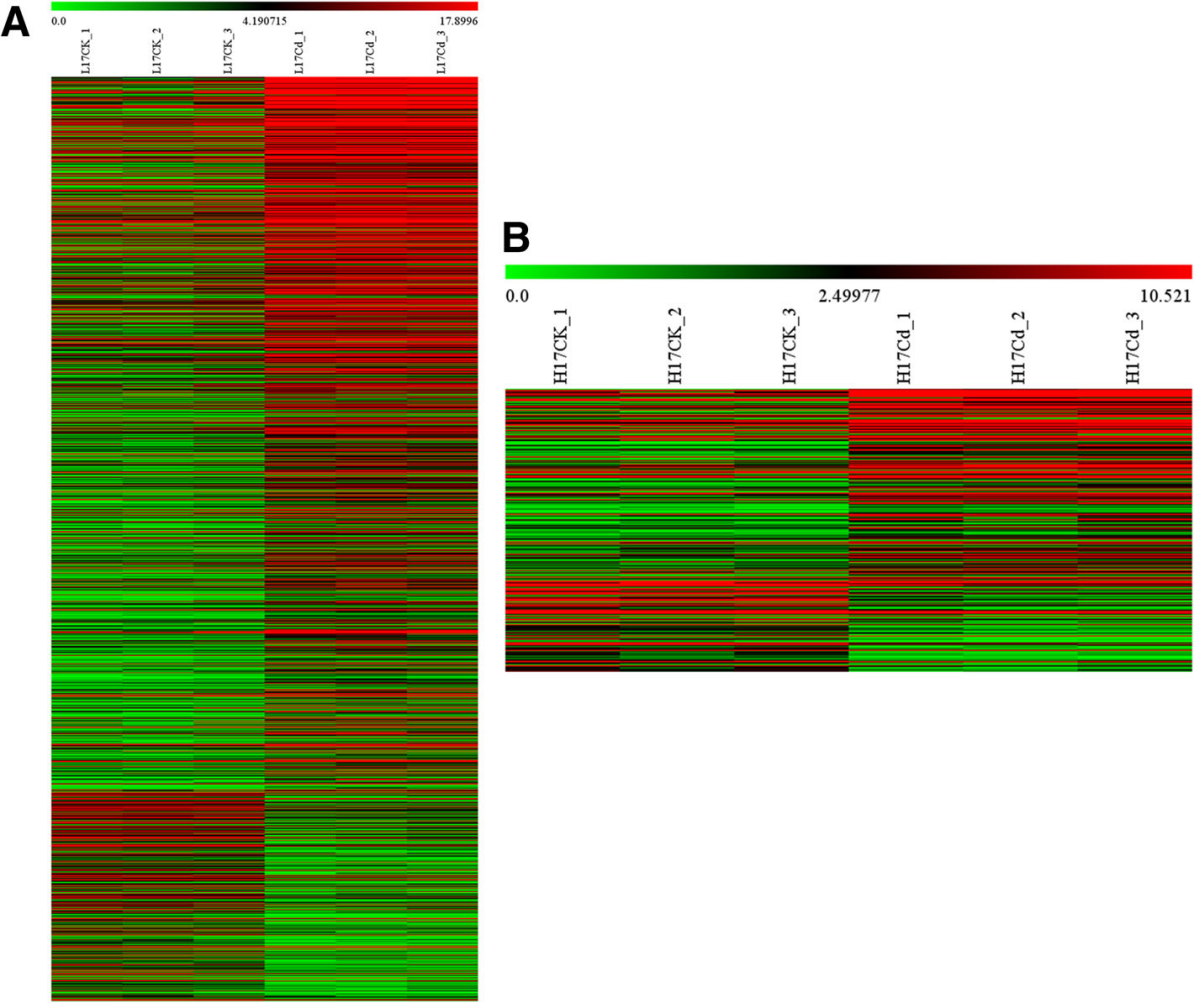
Differentially expressed mRNAs. **a** Heat map of differentially expressed mRNA with fold changes ≥2.0, *P* < 0.05 and false discovery rate (FDR) < 0.05 between L17Cd and L17CK. **b** Heat map of differentially expressed mRNA with fold changes ≥2.0, *P* < 0.05 and FDR < 0.05 between H17Cd and H17CK

After checking the distributions of expression values after normalization and correlation analysis of expression levels between samples (Additional file 2: Figure S3), we next performed differential expression analysis of mRNA transcripts. In the low-Cd-accumulating genotype (L17), a total of 1561 mRNAs were detected to be differentially expressed with fold change > 2.0, *P* < 0.05 (volcano plot filtering) and FDR < 0.05 (Additional file 2: Figure S4, Fig. 2a). Among them, 1208 and 353 mRNAs were upregulated and downregulated (fold change > 2.0, *P* < 0.05 and FDR < 0.05) in L17 roots compared with controls, respectively. In the high-Cd-accumulating genotype (H17), a total of 297 mRNAs were found to be differentially expressed with a fold change > 2.0, *P* < 0.05 (volcano plot filtering) and FDR < 0.05 (Additional file 2: Figure S4, Fig. 2b). Among them, 204 and 93 mRNAs were upregulated and downregulated (fold change > 2.0, *P* < 0.05 and FDR < 0.05) in H17 roots compared with controls, respectively.

### Verification of differentially expressed miRNAs and mRNAs

**Fig. 3:**
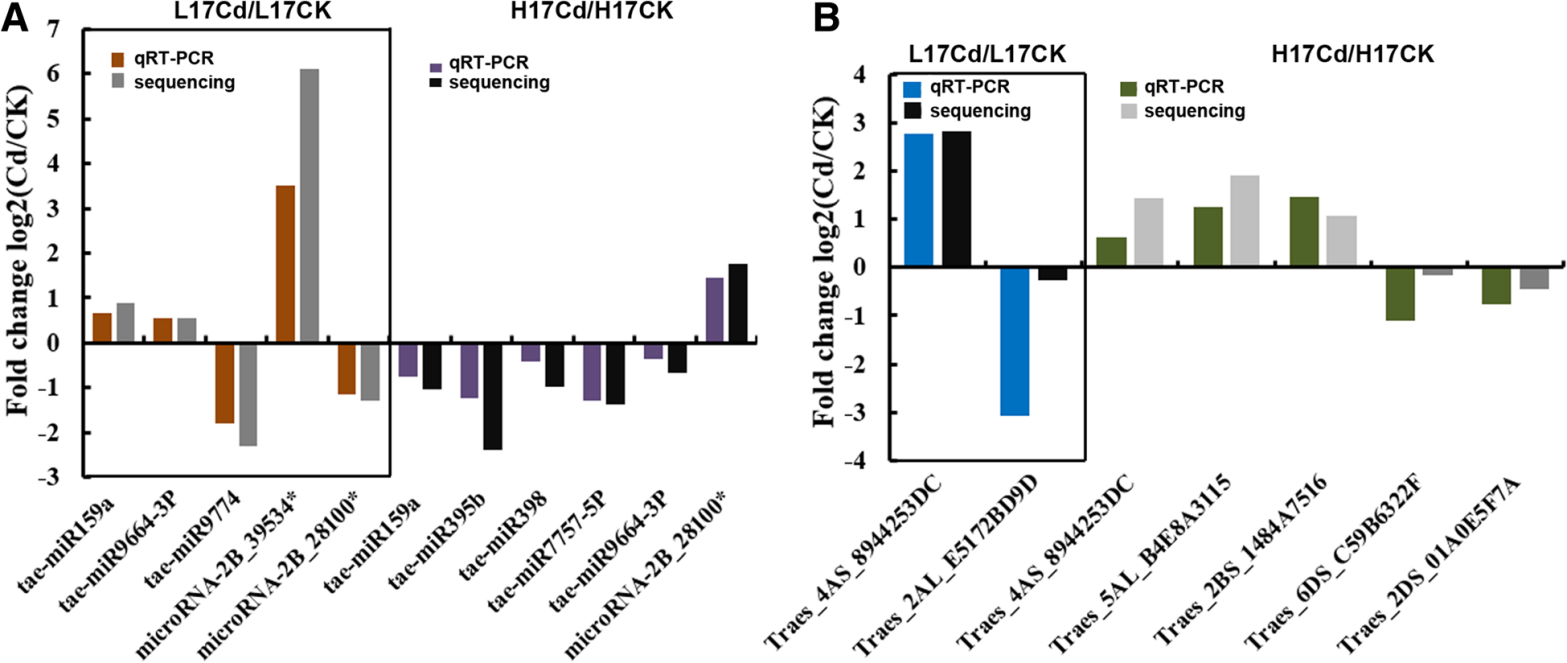
qRT-PCR verification of differential expression analysis. **a** Relative expression levels of five miRNAs shown comparing L17Cd and L17CK (left) and six miRNAs are shown comparing H17Cd and H17CK (right). The heights of the columns stand for the fold changes (log2 transformed) computed from both qRT-PCR and small RNA sequencing data. **b** Relative expression levels of two mRNAs, comparing L17Cd and L17CK (left) and five mRNAs, shown comparing H17Cd and H17CK (right). The heights of the columns stand for the fold changes (log2 transformed) computed from the qRT-PCR and RNA sequencing data. A good correlation of qRT-PCR results and RNA sequencing data are shown through comparing such two results in both cases

To confirm the results of the differential expression analyses, eight Cd-induced miRNAs and six Cd-induced mRNAs were selected for qRT-PCR expression analysis according to their fold change and GO analysis. For microRNAs, compared with L17CK group, tae-miR159a, tae-miR9446-3P, microRNA-2B_39534* were increased in L17Cd group, while tae-miR9774, microRNA-2B_28100* were decreased (Fig. 3a). Compared with H17CK group, microRNA-2B_28100* was increased in H17Cd group, while tae-miR159a, tae-miR395b, tae-miR398, tae-miR7757-5P and tae-miR9664-3P microRNA-2B_28100* (Fig. 3a). For mRNAs, compared with L17CK group, Traes_4AS_8944253DC was increased in L17Cd group, while Traes_2AL_E5172BD9D was decreased (Fig. 3b). Compared with H17CK group, Traes_4AS_8944253DC, Traes_5AL_B4E8A3115 and Traes_2BS_1484A7516 were increased in H17Cd group, while Traes_6DS_C59B6322F and Traes_2DS_01A0E5F7A (Fig. 3b). Hence, the qRT-PCR data verified the small RNA and RNA sequencing results.

### GO and KEGG enrichment analysis

It is well known that miRNAs play essential roles in plant growth and development and also control plant responses to biotic and abiotic stresses by regulating targeted gene expression [23, 40]. Therefore, Gene Ontology (GO) analysis of targeted mRNAs of miRNAs can real the role of differentially expressed miRNAs. Enriched GO categories between L17Cd and L17CK groups, H17Cd and H17CK groups, according to the gene number enriched in GOs, the top 10 biological process (BP), molecular function (MF) and cellular component (CC) for downregulated miRNAs and upregulated miRNAs were shown in (Additional file 2: Figure S5). Overlaying gene sets (Additional file 2: Figure S6) revealed that down-regulated miRNAs in L17 consisted of genes involved in ubiquitin-dependent protein catabolic processes and cell wall organization, whereas up-regulated miRNAs in L17 consist of those involved in protein glycosylation and oxidoreductase activity. Down-regulated miRNAs in H17 consist of those involved in vesicle-mediated transport, and response to heat, whereas up-regulated miRNAs in H17 consist of those involved in photosynthetic electron transport chain and iron ion binding.

For mRNAs, GOs between L17Cd and L17CK groups, H17Cd and H17CK groups, according to the gene number enriched in GOs, the top 10 BP, MF and CC for upregulated mRNAs and downregulated mRNAs were shown in (Additional file 2: Figure S7). Based on the results of Venn diagrams, we found that up-regulated mRNAs in L17 prefer to cell wall macromolecule catabolic process and response to stress, whereas down-regulated mRNAs in L17 prefer to cell wall organization and DNA replication; up-regulated mRNAs in H17 prefer to photosynthesis and nicotianamine biosynthetic process, whereas down-regulated mRNAs in H17 prefer to defense response and metal ion transport (Additional file 2: Figure S6).

**Fig. 4:**
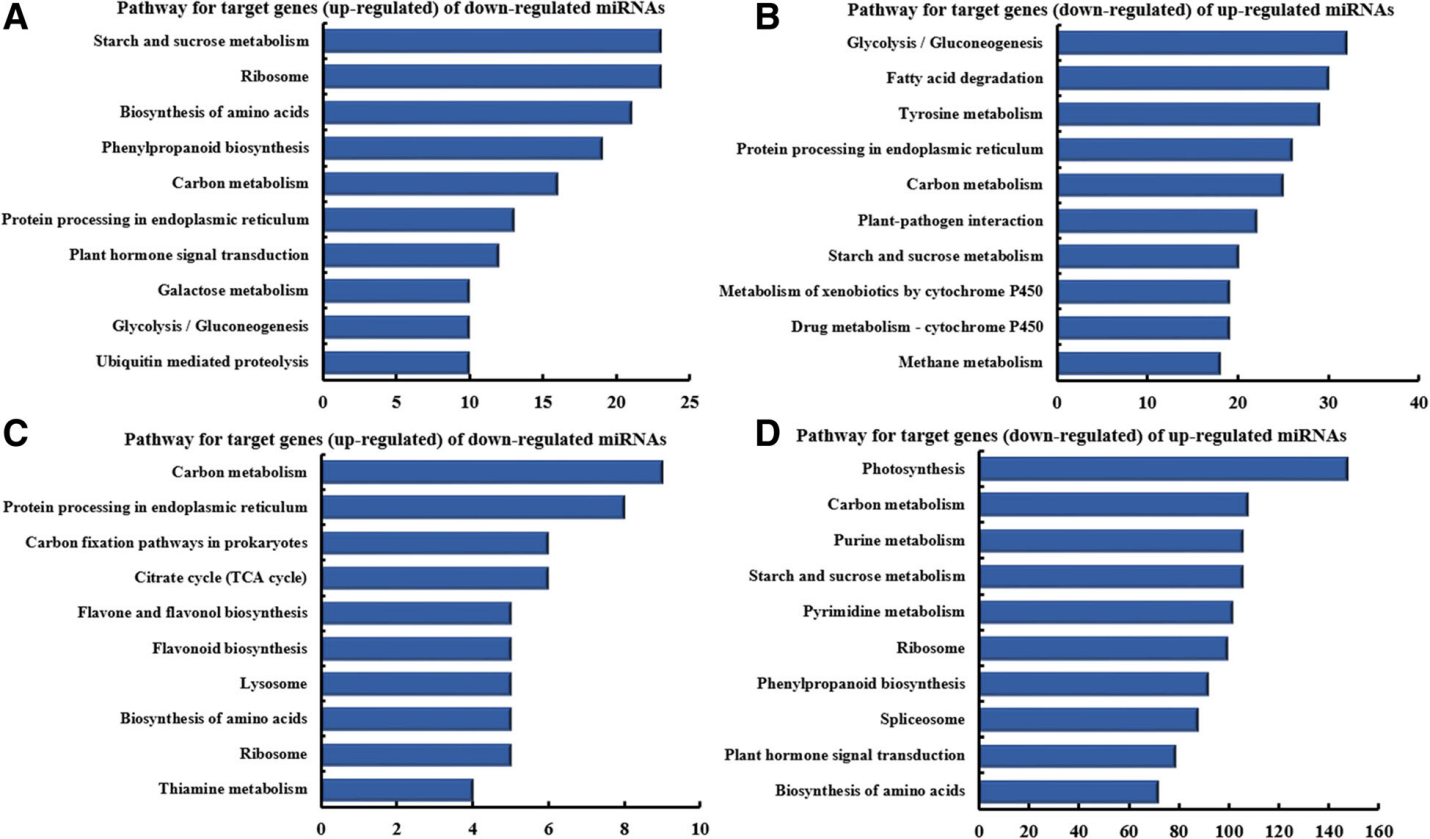
KEGG enrichment analysis of differentially-expressed miRNA-targeted genes. The top 10 pathways enriched in targeted genes are given in response to miRNAs: **a** down-regulated between L17Cd and L17CK; **b** up-regulated between L17Cd and L17CK; **c** down-regulated between H17Cd and H17CK; and **d** up-regulated between H17Cd and H17CK. The x-axis represent the number of genes enriched

**Fig. 5:**
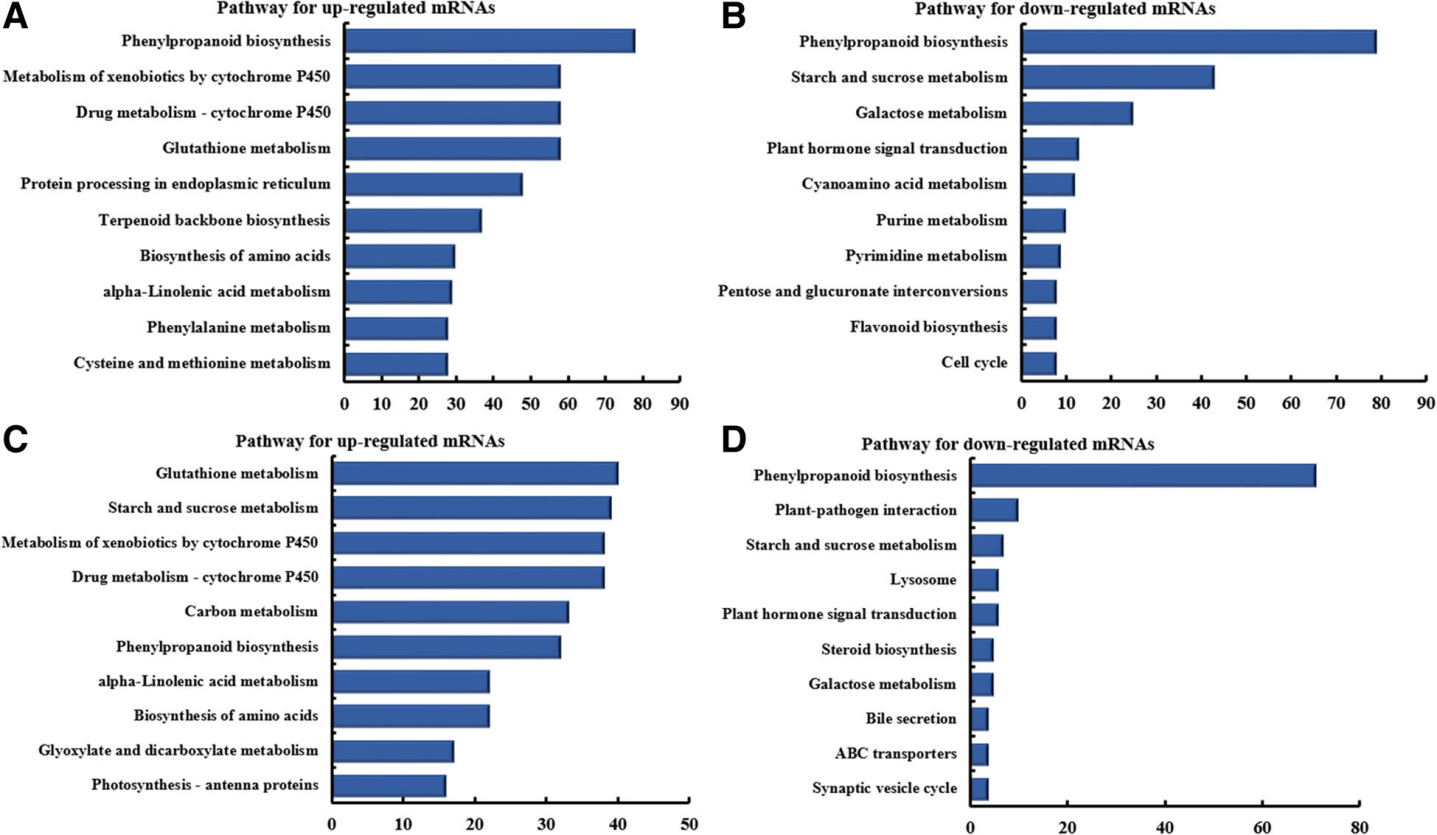
KEGG pathway analysis for differentially-expressed mRNAs. The top 10 pathways enriched in differentially expressed mRNAs are given for the contrasts: **a** up-regulated between L17Cd and L17CK; **b** down-regulated between L17Cd and L17CK; **c** up-regulated between H17Cd and H17CK; and **d** down-regulated between H17Cd and H17CK. The x-axis represent the gene number enriched in pathways

Analysis of pathway enrichment for miRNAs between L17Cd and L17CK groups revealed a diverse set of processes responsive to Cd (Fig. 4a, b). Among these pathways, starch and sucrose metabolism was the most prominent pathway targeted by downregulated miNAs (Fig. 4a), whereas glycolysis / gluconeogenesis was the top enriched pathway for up-regulated miRNAs (Fig. 4b). In the H17Cd and H17CK contrasts we found that carbon metabolism was the most prominent pathway targeted by downregulated miNAs (Fig. 4c), whereas photosynthesis was the most prominent pathway targeted by upregulated miNAs (Fig. 4d). For mRNAs differentially regulated between L17Cd and L17CK groups, the top 10 pathways associated with up- or down-regulated mRNAs are given in Fig. 5. Among these pathways, phenylpropanoid biosynthesis was the top pathway in upregulated mRNAs (Fig. 5a), whereas starch and sucrose metabolism was the top one in downregulated mRNAs (Fig. 5b). Pathways enriched in the H17Cd and H17CK groups are given in Fig. 5c, where glutathione metabolism was most prominent. On the other hand, phenylpropanoid biosynthesis was the most prominent in down-regulated mRNAs (Fig. 5d).

Our results are complement previous work in other systems. Feng et al. [41] found enrichment of genes differentially-expressed in response to Cd in two sweet sorghum genotypes. Enriched in the high Cd-accumulating sweet sorghum genotype were genes that *Sb04g033320*, *Sb04g033330,* and *Sb01g012440*. Enriched in the low Cd-accumulating sweet sorghum genotypes were genes that *Sb02g006960*, *Sb01g043400*, and *Sb10g022390*. Other studies have indicated that transporters for essential elements such as Fe^2+^, Zn^2+^ and Ca^2+^ are involved in Cd uptake and transport [42]. Numerous heavy metal transporters, such as ATP-binding cassette transporters (ABC), natural resistance-associated macrophage proteins (Nramp) and heavy metal ATPases (HMA) participate in the acquisition, distribution, and homeostasis of Cd in plants [27, 43, 44]. The MAPK signaling pathway is also part of the miRNA-target gene network. MAPK signaling cascades are one of the most conserved pathways in plants and are known to modulate plant response to abiotic stress [45]. Taken together, these studies and ours indicate that the interaction effects of multiple miRNAs are involved in the regulation of diverse physiological systems to relieve the toxicity induced by Cd.

### Genes related to transport

Between L17Cd and L17CK groups 223 up-regulated genes and 183 down-regulated genes are classified as transporters based on functional annotation. Between H17Cd and H17CK, 162 up-regulated genes and 134 down-regulated genes were transporter genes according to the functional annotation of the differentially expressed genes (Additional file 3). Of these, Traes_2AL_9B175F3DA, Traes_2AS_95611CAD2, Traes_2DL_D5F2272A9, Traes_2BS_3212EB7DF, Traes_7BL_39745BF3F, Traes_7DL_44DA42073, Traes_2DL_2A1D45D02, Traes_3DL_4EE343C68, Traes_5BL_4BCE2DBEF, Traes_2BS_3212EB7DF, Traes_3AS_CBB5F7EB6, Traes_4DL_560FD0832, Traes_7AL_3E508AA9C, Trae_7DL_A5269C73F, Trae_7AL_8304348B7 and Trae_7BL_A4BE6BD10 are related to metal ion.

### Genome-wide identification and phylogenetic analysis of HMA gene in wheat

**Fig. 6:**
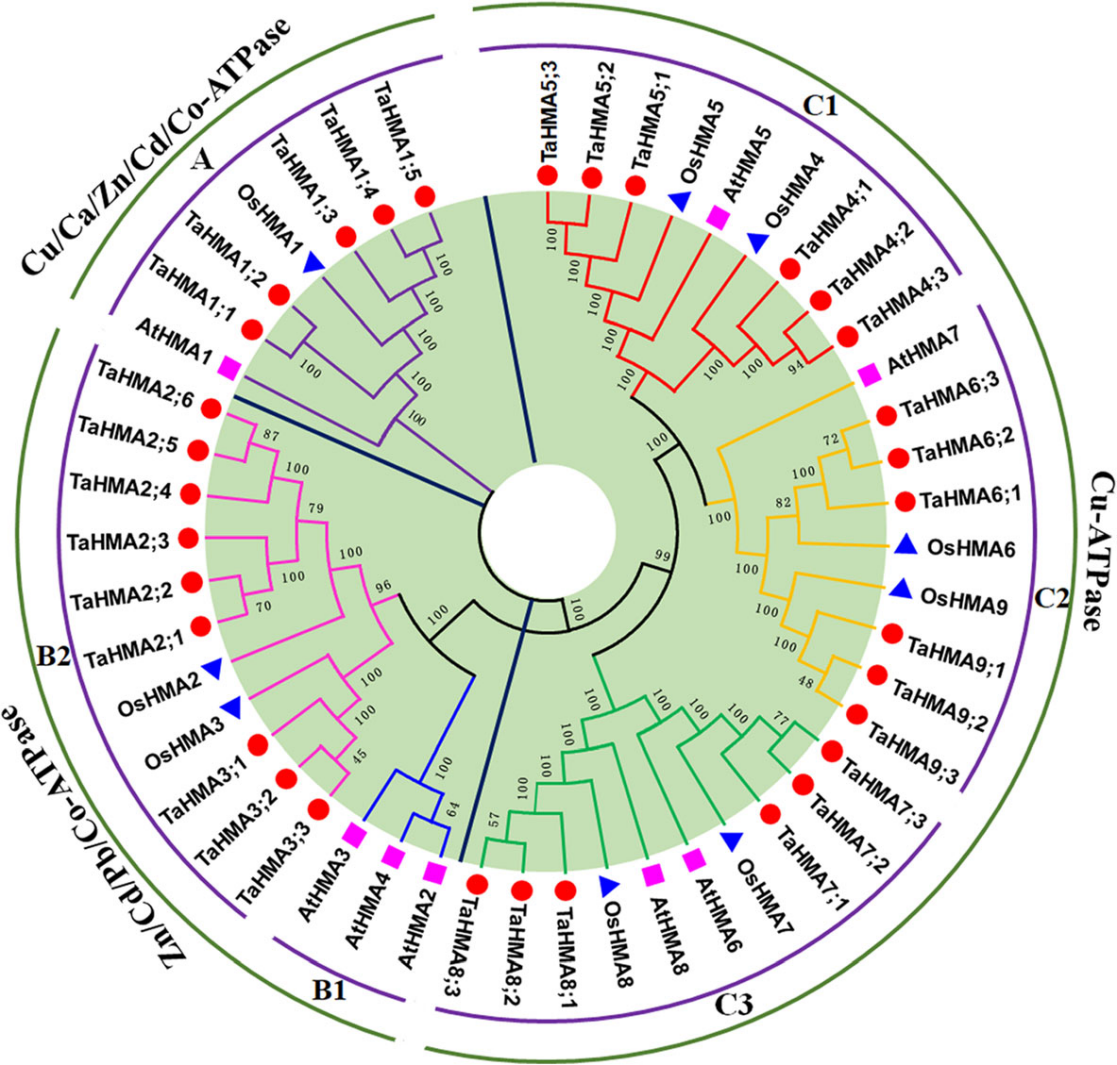
Neighbour-Joining phylogenetic tree of HMA proteins from wheat (Ta), Aradidopsis (At), and rice (Os). HMA proteins were used to establish the phylogenetic tree with MEGA6.0. TaHMA proteins are marked in red. An unrooted Neighbour-Joining analysis was performed with pairwise deletion and Poisson correction

Through the availability of the wheat genome sequence, it is now possible to identify all the HMA family members in wheat. Here we identify a total of 32 HMA members in the wheat genome (Table 4) based on the genome sequence. We further performed a BLASTN search against the wheat expressed sequence tag (EST) using these HMAs queries to verify the existence of these wheat HMAs. Results showed that most of the TaHMAs’ we found in the genome sequence were supported by EST hits. Among the supported TaHMA genes, TaHMA1;4 has the largest hits of ESTs, with 61, followed by TaHMA6;3 with 53. The lengths of these predicted TaHMA proteins varied from 419 aa to 1702 aa with the putative molecular weights (Mw) ranging from 44.07 to 185.21 kDa and theoretical isoelectric points (PI) ranging from 5.23 to 8.21. Eight HMA proteins are known in *A.thaliana* and nine in *O.sativa*, which range from 542 aa to 1171aa and from 792 aa to 1060 aa, respectively [46]. Using full-length HMA protein sequences from *Triticum aestivum, A.thaliana* and *O.sativa,* we constructed a phylogentic tree to determine the phylogenetic relationship between HMA proteins from *Triticum aestivum* and those from other species. The HMA gene families could be divided into three groups: the Cu/Ca/Zn/Cd/Co-ATPases group, the Zn/Cd/Pb/Co-ATPase group, and the Cu-ATPase group [47]. TaHMA1;1-TaHMA1;5 belong to the Cu/Ca/Zn/Cd/Co-ATPases group, TaHMA2;1-TaHMA2;6, TaHMA3;1-TaHMA3;2 belong to the Zn/Cd/Pb/Co-ATPase group, TaHMA4;1-TaHMA4;3, TaHMA5;1-TaHMA5;3, TaHMA6;1-TaHMA6;3, TaHMA7;1-TaHMA7;3, TaHMA8;1-TaHMA8;3, TaHMA9;1-TaHMA9;3 belong to the Cu-ATPase group (Fig. 6). To our knowledge, it is first genome-wide analysis of the HMA family in wheat.

**Table 4:** Identified putative wheat HMA genes

HMAs	Subfamily gene name	Ensemble Wheat Gene ID	Chromosome location	Length (bp)	Exons	Introns	Amino acid length (aa)	PI	MW (kDA)	EST count
TaHMA1;1	TaHMA1	Trae_5AL_C89EEBE50	TGACv1_scaffold_375043_5AL:69226–81,875	12,650	16	15	790	6.31	84.44	2
TaHMA1;2		Trae_5BL_F83C809F0	TGACv1_scaffold_405147_5BL:88295–97,375	9081	13	12	480	8.21	51.96	2
TaHMA1;3		nd	TGACv1_scaffold_556453_7AL:106656–112,341	5686	8	7	419	6.56	44.07	20
TaHMA1;4		Trae_7BL_041308E74	TGACv1_scaffold_578186_7BL:25106–34,662	9557	14	13	832	6.90	88.37	61
TaHMA1;5		Trae_7DL_FBCC75BBC	TGACv1_scaffold_603768_7DL:23146–31,623	8478	12	11	748	6.98	78.96	39
TaHMA2;1	TaHMA2	Trae_7DL_A5269C73F	TGACv1_scaffold_602651_7DL:105511–113,285	7742	10	9	1003	6.53	108.33	34
TaHMA2;2		Trae_7AL_8304348B7	TGACv1_scaffold_556712_7AL:90458–108,176	17,719	9	8	985	6.29	106.46	21
TaHMA2;3		Trae_7BL_A4BE6BD10	TGACv1_scaffold_579416_7BL:14127–24,069	9282	10	9	1023	6.51	110.32	37
TaHMA2;4		Trae_7BL_0CF58CF4E	TGACv1_scaffold_577252_7BL:118443–123,897	5455	9	8	766	7.83	81.09	3
TaHMA2;5		Trae_7AL_6AE850114	TGACv1_scaffold_557470_7AL:61198–66,965	5768	10	9	939	7.11	99.55	6
TaHMA2;6		Trae_7DL_EED4BED66	TGACv1_scaffold_603863_7DL:43200–49,689	5572	8	7	800	6.44	84.37	6
TaHMA3;1	TaHMA3	Trae_5BL_D6C3DC326	TGACv1_scaffold_404346_5BL:222670–226,813	4108	6	5	829	6.05	86.82	3
TaHMA3;2		nd	TGACv1_scaffold_375473_5AL:29654–33,170	3517	6	5	816	6.29	85.66	2
TaHMA3;3		nd	TGACv1_scaffold_435190_5DL:5098–8725	3628	6	5	853	6.12	89.09	3
TaHMA4;1	TaHMA4	Trae_6DS_26C5A0A44	TGACv1_scaffold_543118_6DS:56997–63,045	6049	10	9	687	6.01	73.46	30
TaHMA4;2		Trae_6BS_A8B960E60	TGACv1_scaffold_515718_6BS:7391–13,506	6116	9	8	980	5.44	105.63	29
TaHMA4;3		Trae_6AS_6F306F27E	TGACv1_scaffold_485988_6AS:25642–29,385	3744	7	6	687	6.01	73.47	25
TaHMA5;1	TaHMA5	Trae_2DL_51FF05F66	TGACv1_scaffold_157956_2DL:22600–32,475	9876	6	5	1000	5.65	108.18	9
TaHMA5;2		Trae_2AL_D0EABF355	TGACv1_scaffold_099043_2AL:1541–7440	5900	6	5	1011	5.66	109.11	9
TaHMA5;3		Trae_2BL_19B3E60AA	TGACv1_scaffold_130949_2BL:4724–10,512	5789	6	5	994	5.88	107.49	9
TaHMA6;1	TaHMA6	Trae_6BS_9A12C2A1D	TGACv1_scaffold_513141_6BS:286354–292,595	6242	9	8	974	5.73	104.50	50
TaHMA6;2		Trae_6DS_9FA053DF8	TGACv1_scaffold_542481_6DS:33526–39,252	5727	9	8	1042	5.53	111.76	50
TaHMA6;3		Trae_6AS_9321C1C5B	TGACv1_scaffold_486033_6AS:36396–41,875	5480	9	8	997	5.23	106.85	53
TaHMA7;1	TaHMA7	Trae_7AS_766146E70	TGACv1_scaffold_569059_7AS:131450–140,663	9214	17	16	947	7.26	99.55	22
TaHMA7;2		Trae_7BS_8EC4B41E4	TGACv1_scaffold_592600_7BS:48974–58,635	9662	17	16	952	7.23	100.02	26
TaHMA7;3		Trae_7DS_04F16455B	TGACv1_scaffold_622783_7DS:19856–29,376	9521	17	16	952	6.98	99.94	26
TaHMA8;1	TaHMA8	Trae_4BL_89775421A	TGACv1_scaffold_640736_U:185896–195,006	9111	17	16	903	6.07	94.48	37
TaHMA8;2		Trae_4AS_622EEFE10	TGACv1_scaffold_306691_4AS:30689–37,419	6731	17	16	900	5.85	94.16	37
TaHMA8;3		Trae_4DL_385639507	TGACv1_scaffold_344605_4DL:1306–16,767	15,462	20	19	1702	6.93	185.21	30
TaHMA9;1	TaHMA9	Trae_7AL_7A2639A1B	TGACv1_scaffold_558946_7AL:1236–7780	6545	9	8	1001	5.29	106.89	17
TaHMA9;2		Trae_7DL_DF97DD324	TGACv1_scaffold_604439_7DL:28926–35,180	6255	9	8	1001	5.40	106.78	18
TaHMA9;3		Trae_7BL_EFF0E2E31	TGACv1_scaffold_579154_7BL:35573–41,675	6103	9	8	1001	5.28	106.77	16

### Chromosomal location, gene structure and conserved motif analysis of TaHMAs

Chromosome localization analysis revealed that 31 of the TaHMA genes were distributed on 2A, 2B, 2D, 4A, 4D, 5A, 5B, 5D, 6A, 6B, 6D, 7A, 7B and 7D, of which 7A, 7B, 7D contained the greatest number of HMA genes, while one TaHMA did not have a corresponding chromosome (Table 4). To further understand the features of the genes of the HMA family, the exon/intron structures of TaHMA genes were analyzed. As shown in Table 1, the number of introns varied from 6 to 20. TaHMA8;3 has the largest number of introns (19), with TaHMA3;1, TaHMA3;2, TaHMA3;3, TaHMA5;1, TaHMA5;2 and TaHMA5;3 having the fewest introns (5). Although the exons varied among TaHMA genes, the most closely related members have similar gene structures. To predict and verify domains in the TaHMAs proteins, we used the Multiple EM for Motif Elicitation (MEME) motif search tool. Ten corresponding consensus motifs were detected (Additional file 2: Figure S8, Table 5). The number of motifs varied among the TaHMA proteins. Motif 2, motif 6, motif 8, motif 9 and motif 10 were observed in all TaHMA proteins. Motif 1 and motif 3 were observed in all TaHMA proteins except TaHMA1;3.

**Table 5:** Motif sequences for HMAs identified by MEME analysis

Motif	Width	Multilevel consensus sequence
1	50	LNLBGYJHVRATKVGSNSALAKIVRLVEEAQMSKAPVQRLADKVAKYFVP
2	41	TPTAVMVATGVGARRGVLIKGGDVLESLANIKAIAFDKTGT
3	50	VGDVIKVLPGEKVPVDGVVVDGRSHVBESMLTGESAPVAKZVGSEVIGGT
4	50	AIGSGTAVAIEAADVVLMSNBLEDVPTAIDLSRKTFRTIRQNYVWAVAYN
5	41	KFFEESGMJVFFFLLGKYLEVLAKGKASDAMSKLMELAPET
6	29	ELQKRGGPVAMVGDGINDAPALAAADVGM
7	50	IVGIPVAAGALFPFTGFRLPPWLAGACMAFSSVSVVCSSLLLRLYKKPRH
8	26	SMGIKSVMLTGDNWAAAQAVAKZVGI
9	29	ELLYLVASAESNSEHPLAKAIVEYAQSFS
10	29	EEVADFEILPGEGVYAEIDGKKVLVGNKR

Motif numbers corresponded to the motifs in Additional file 2: Figure S8

### Tissue-specific expression patterns of TaHMA genes

**Fig. 7:**
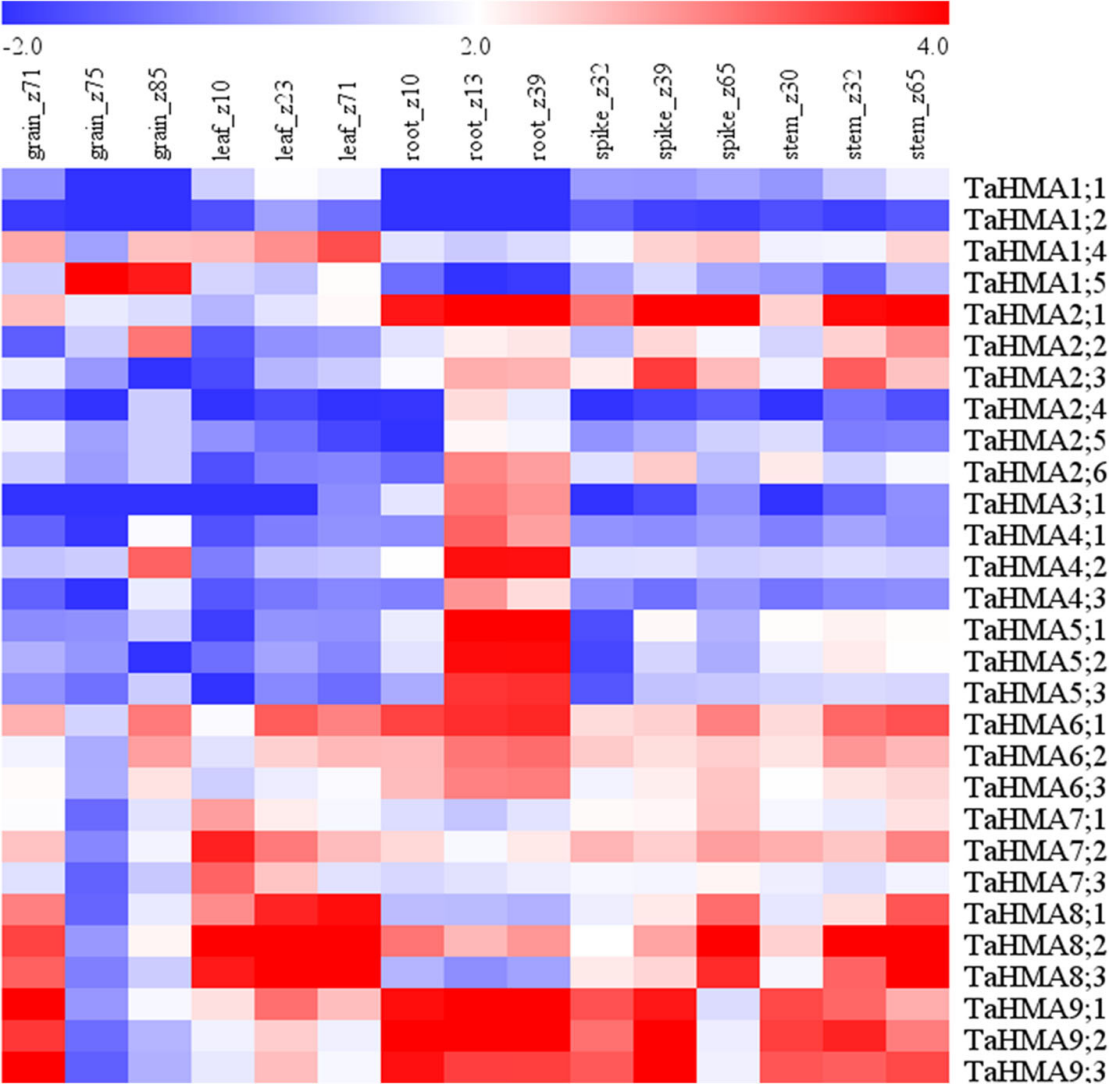
Heat map of the expression profiles of 29 TaHMA genes in five different tissues (grain, leaf, root, spike and stem). Log2 transformed FPKM values are represented. The red or blue colors indicate higher or lower relative abundance, respectively. Z represent Zadoks scale, a decimal code for the growth stages of cereals. *P*-value< 0.05 were regarded as statistically significant

Using available RNA-seq data (the wheat expression database, http://wheat.pw.usda.gov/WheatExp/) for five different tissues, the tissue specificity of the TaHMA genes was investigated to focus on the temporal and spatial expression patterns and putative functions of HMA genes in the growth and development. According to FPKM values, we found that the expression levels of the TaHMAs varied significantly in different tissues (Fig. 7). Most HMAs were found to be expressed in five detected organs. TaHMA1;1, TaHMA1;2, TaHMA2;5 and TaHMA4;3 showed weak expression in all tissues, while TaHMA6;1, TaHMA9;1, TaHMA9;2 and TaHMA9;3 had strong expression. TaHMA1;4, TaHMA2;1, TaHMA2;2, TaHMA2;4, TaHMA2;5, TaHMA7;1, TaHMA7;2, TaHMA7;3, TaHMA8;1, TaHMA8;2 and TaHMA8;3 were differentially expressed in 5 organs.

### Expression pattern of TaHMA genes under cd stress

**Fig. 8:**
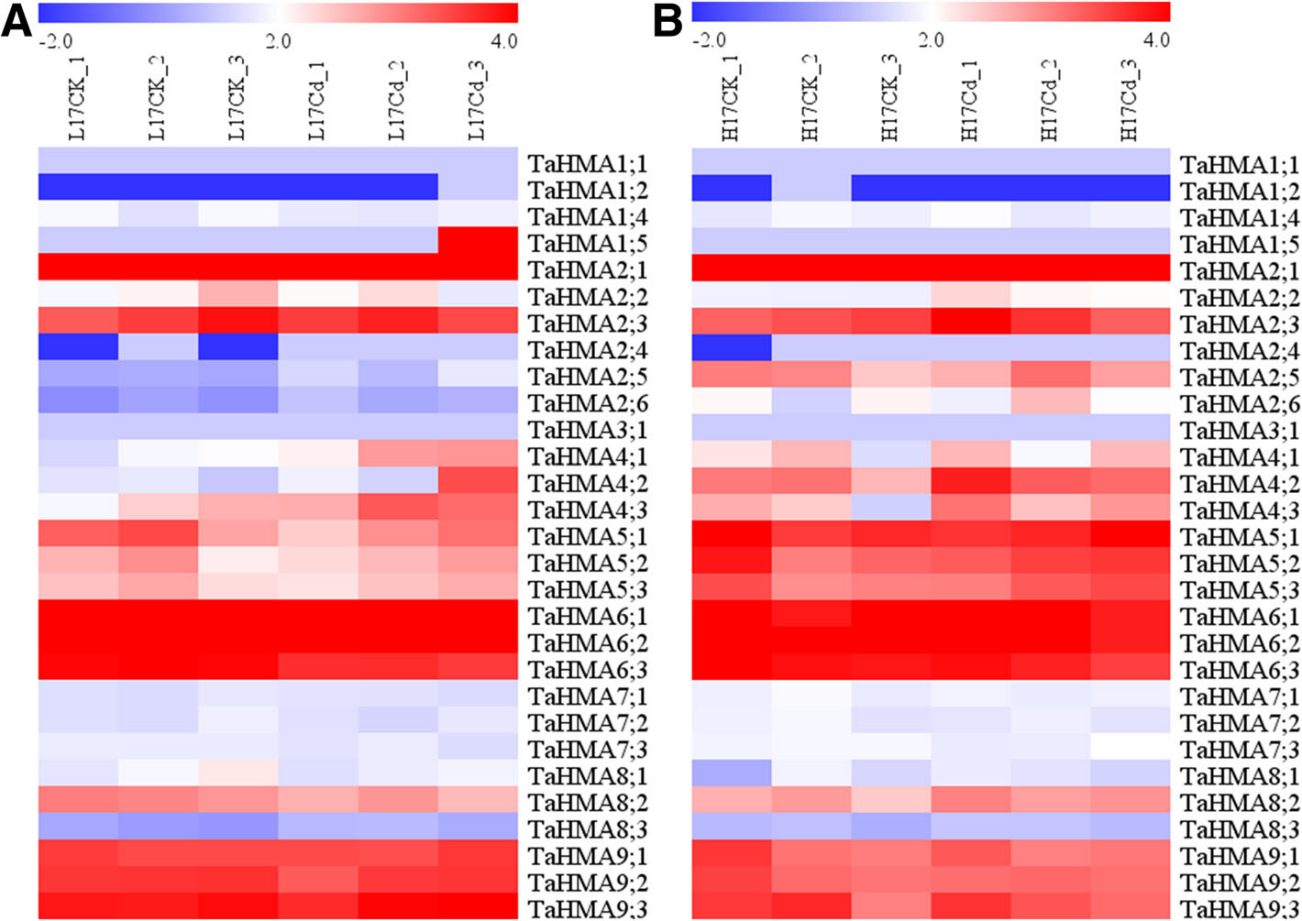
Heat map of the expression profiles of TaHMA genes under Cd treatment. **a** Expression profiles of TaHMA genes in L17Cd and L17CK groups. **b** Expression profiles of TaHMA genes in H17Cd and H17CK groups. Log2 transformed FPKM values were used to create the heat map. Red or blue indicates higher or lower relative abundance, respectively

To characterize potential roles of TaHMA genes in response to Cd stresses, expression of all TaHMA genes in response to Cd stress were investigated using our RNA sequencing data. Overall, the 32 wheat HMA genes exhibited diverse expression patterns under Cd challenge. In L17, the expression levels of TaHMA1;2, TaHMA1;4, TaHMA1;5, TaHMA2;2, TaHMA5;1, TaHMA6;1, TaHMA6;3, TaHMA7;1, TaHMA7;2, TaHMA7;3, TaHMA8;1, TaHMA8;2 and TaHMA9;2 were down-regulated under Cd stresses, while, the expression levels of TaHMA2;1, TaHMA2;3, TaHMA2;5, TaHMA2;6, TaHMA4;1, TaHMA4;2, TaHMA4;3, TaHMA5;2, TaHMA5;3, TaHMA6;2, TaHMA8;3 and TaHMA9;1, TaHMA9;3 were up-regulated (Fig. 8a). In H17, the expression levels of TaHMA1;5, TaHMA5;1, TaHMA6;1, TaHMA6;2, TaHMA6;3, TaHMA7;2, TaHMA7;3, TaHMA9;1, TaHMA9;2 and TaHMA9;3 were down-regulated under Cd stresses, while, the expression levels of TaHMA1;2, TaHMA1;4, TaHMA2;1, TaHMA2;2, TaHMA2;3, TaHMA2;5, TaHMA2;6, TaHMA4;1, TaHMA4;2, TaHMA4;3 TaHMA5;2, TaHMA5;3, TaHMA7;1 TaHMA8;1, TaHMA8;2 and TaHMA8;3 were both up-regulated (Fig. 8b).

### Integration analysis miRNAs and TaHMAs

**Fig. 9:**
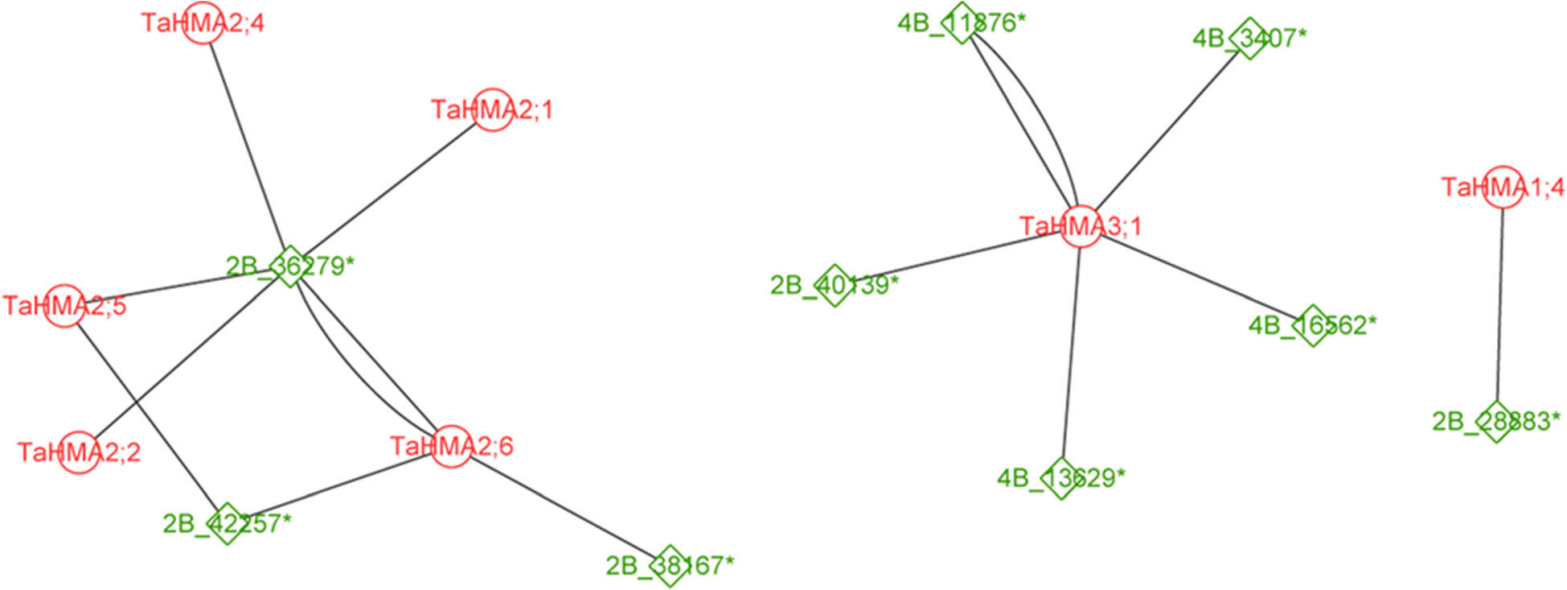
Relationships between miRNAs and TaHMAs given by co-expression network analysis

To explore the regulatory association between miRNAs and TaHMAs, microRNA-TaHMAs we performed a expression network analysis using targetfinder in plant [48] which was shown using Cytoscape v3.6 (http://www.cytoscape.org/). Results showed that microRNA-2B_36279* can regulate TaHMA2;1, TaHMA2;2, TaHMA2;4, TaHMA2;5 and TaHMA2;6, while microRNA-4B_11876*, microRNA-4B_3407*, microRNA-4B_16562*, microRNA-4B_13629* and microRNA-2B_40139* can regulate TaHMA3;1 (Fig. 9), and microRNA-2B_28883* can regulate TaHMA1;4. To our knowledge, it is the first time to perform microRNA-TaHMA regulatory network in wheat.

### Response of selected TaHMA genes to exogenous cd stress

**Fig. 10:**
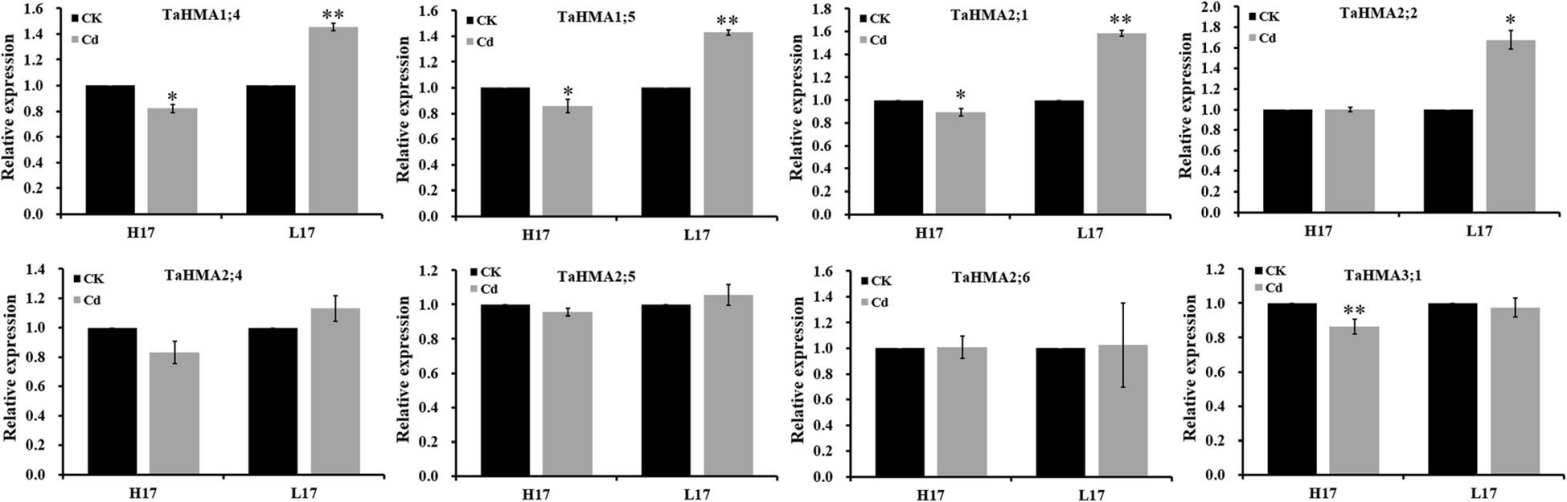
Relative expression of TaHMAs in high- (H17) and low- (L17) cadmium-accumulating wheat cultivars under Cd stress. Relative expression levels of TaHM1-TaHMA3 were analyzed using qRT-PCR. H17 and L17 wheat samples were treated with 100 μM Cd. H17 and L17 wheat samples growing under Cd-free conditions were used as controls

HMAs have been implicated in the transmembrane transport of metals, such as Zn, Cd, Pb and Cu in different plant species [46, 49–51]. Therefore, we were interested to determine the response of select HMA genes under Cd stress. We thus selected eight *TaHMA* genes (*TaHMA1;4*-*TaHMA3;1*) belonging to the Cu/Ca/Zn/Cd/Co-ATPase group and the Zn/Cd/Pb/Co-ATPase group to determine their relative expression levels in roots under Cd stress using qRT-PCR. Results showed that relative expression levels of TaHMA1;4, TaHMA1;5, TaHMA2;1 and TaHMA3;1 were significantly down-regulated under Cd treatment compared with those under control conditions in high-accumulating wheat genotype (H17), while relative expression levels of TaHMA1;4, TaHMA1;5, TaHMA2;1 andTaHMA2;2 were significantly up-regulated under Cd treatment compared with those under control conditions in low-accumulating wheat genotype (L17) (Fig. 10). Relative expression of TaHMA1;5 in H17, TaHMA2;1 in L17 was consistent with our transcriptome sequencing data. Interestingly, expression data from other TaHMA genes in H17 and L17 treated with Cd were not consistent with sequencing data. A possible explanation for this inconsistence is the number of samples used on this experiment. Previous work indicated that AtHMA1 is responsible for Cu to the stroma, exporting Zn2+ form the chloroplast, or as a Ca^2+^/heavy metal transporter to the intracellular organelle [52–54]. AtHMA2 and AtHMA4 in *A.thaliana* are involved in xylem loading of Zn as well as Cd [55–59]. Compared with wild-type plants, plants overexpressing AtHMA3 exhibited a 2–3-fold increase in Cd accumulation [28]. AtHMA5 is responsible for the Cu translocation from roots to shoots or Cu detoxification of roots [60, 61]. AtHMA6 (also known as PAA1) is involved in transporting Cu over the chloroplast envelope, whereas AtHMA8 (PAA2) most likely transports Cu into the thylakoid lumen to supply plastocyanin [62, 63]. AtHMA7 has been proposed to delivery Cu to ethylene receptors and Cu homeostasis in the seedlings [64, 65]. Among rice HMA characterized, OsHMA1 transports Zn and Cd [66], OsHMA2 is responsible for Cd accumulation [67], OsHMA5 is involved in loading Cu to the xylem of the roots and other organs [68], and OsHMA9 is responsible for transporting Zn, Cu, Pb and Cd [69]. In wheat, TaHMA2 is a plasma membrane-localized Zn/Cd transporter that pumps Zn/Cd into apoplast [26]. Our results indicated that Cd stress induced the up-regulation of TaHMA2;1 in L17.

## Conclusions

We present here a broad range of genome-wide data surrounding Cd response in contrasting wheat lines. A total three known and 19 novel differentially expressed microRNAs (DEMs), and 1561 differentially expressed genes (DEGs) were found in L17 after Cd treatment. In H17 we found 12 known and 57 novel DEMs, 297 Cd-induced DEGs. Moreover, we present the identification of 32 TaHMA genes in wheat. Finally, we provide microRNA-TaHMA expression networks that suggest that miRNAs can regulate TaHMAs which need to be validated experimentally in future. Taken together, our results indicate that microRNAs may play important roles in wheat under Cd stress through regulating target genes such as TaHMA2;1. This information will be useful for screening and breeding low-Cd accumulation wheat genotypes.

## Methods

### Study site and wheat germplasms

The Shifang heavy industry and Cd-polluted area was selected as our study site in the northeast of Sichuan province. Low grain Cd accumulative wheat cultivars were selected from the Cd-polluted area as suitable for planting in Cd pollution farmland. A total of 185 wheat germplasms (provided by Chengdu Institute of Biology, Chinese Academy of Sciences) were chosen to include wide genetic diversity. Detailed information of wheat cultivars were shown in Additional file 1: Table S1. The 185 wheat germplasms could be divided into two groups, including 181 bread wheat (*Triticum aestivum* L.) and 4 durum wheat (*Triticum turgidum* L. var. durum) groups. The 181 bread wheats were further divided into three groups, including 52 Chuanyu wheat cultivars (Chuanyu group), 56 International Maize and Wheat Improvement Center (CIMMYT) materials (CIMMYT group) and 73 Chinese modern cultivars (CMC group).

### Plant growth conditions and cd treatment

A low-Cd-accumulating cultivar (Chuanyu 17, namely L17) and its sib-line H17 (a high-Cd-accumulating cultivar) from Chuanyu group were used in the study. Seeds were sterilized by soaking in 2% H_2_O_2_ for 10 min and fully rinsed with deionized water. After sterilizing, the seeds were soaked in deionized water at room temperature for 6 h, and then planted hydroponically in Hoagland solution in growth chambers at 24 ± 2 °C and 50% relative humidity with a photoperiod of 16 h light/8 h dark. One-week-old seedlings were treated with 0 (CK) and 100 μM CdCl_2_ for 24 h (Cd). Roots from the plants of similar size were harvested separately and washed three times with deionized water. Roots from three biological replicates were frozen in liquid nitrogen immediately and stored at − 80 °C for small RNA and transcriptome sequencing.

### Determination of grain and root cd concentrations

For wheat grain, whole grain was ground into flour (< 1 mm). Flour was dried at 80 °C for 12 h and then digested by HNO_3_ and H_2_O_2_ in a microwave digester. Roots from three plants as replicates for each of CK and Cd treatments were dried at 70 °C to a constant weight and then also digested by HNO_3_ and H_2_O_2_ in a microwave digester. All grain samples were processed together with quality controls. Cd concentration was measured using inductively couple plasma-mass spectrometry (ICP-MS, NexIONTM 300, USA) following the manufacturer’s instructions [70]. A Certified Reference material (CRM; GBW10020, provided by the National Research Center for CRM, China) was applied to assess the precision of the analytical procedures for plant material.

### RNA extraction

One week-old L17 and H17 seedlings were treated with 0 (CK) and 100 μM CdCl_2_ (Cd) for 24 h. Then, roots of 12 seedlings (each treatment for three biological replicates) were collected for RNA extraction. Total RNA were extracted using the TRIzol Reagent (Invitrogen, USA) following the manufacturer’s protocol. RNA integrity was evaluated using the Agilent 2100 Bioanalyzer (Agilent Technologies, Santa Clara, CA, USA), and each sample had an RNA integrity number (RIN) > 8.0.

### Small RNA sequencing and analysis

cDNA libraries were built using SuperScript II Reverse Transcriptase basing on the manufacturer’s instructions. Resultant libraries were then sequenced on the Illumina HiSeqTM 2500 sequencing platform (OEbiotech Company, Shanghai, China). Raw reads were converted into sequence data by base calling. After reads with low-quality, 5′ primer contaminants and ploy (A), without 3’adapter and insert tag, shorter than 15 nt and longer than 41 nt from the raw reads were filtered, the clean reads were obtained and mapped against the wheat genome. Clean reads were annotated with miRBase (version 21.0, http://www.mirbase.org/) to identify know miRNAs using Bowtie software. To identify novel miRNAs, the remaining unannotated clean reads that could be aligned to the genome were analyzed by miRDeep2 and RNAfold. The criteria for designation as a novel miRNA were as follows: length of 18-24 nt, precursors with a signature hairpin structure and the formation of maturation achieved by Dicer, minimum free energy of precursors of less than 18 kcal/mol, a minimum support number for the maturity sequence of precursors of at least 5, and potential miRNA sequence with less than 3 nt mispairing in the sequence of the mature and perfectly matched middle sequence. Differentially expressed miRNAs (DEMs) were identified with the threshold of *p* value < 0.05. While the p value was calculated with the DEG algorithm in the R package. The targets of DEMs were predicted by using targetfinder [48].

### Transcriptome sequencing

cDNA libraries were constructed using the TruSeq Stranded mRNA LTSample prep kit (Illumina, Sam Diego, CA, USA) according to the manufacturer’s instructions. Resultant libraries were sequenced on the Illumina HiSeqTM 2500 sequencing platform (OEbiotech Company, Shanghai, China). Raw reads were processed using NGS QC toolkit. The reads containing poly-N and the low quality reads were removed to obtain the clean reads. Clean reads were then mapped to wheat reference genome (ftp://ftp.ensemblgenomes.org/pub/plants/release-28/fasta/triticum_aestivum/dna/Triticum_aestivum.IWGSC1.0+popseq.28.dna.toplevel.fa.gz) [71] using hisat2. Fragments per kilobase of transcript sequence per million base pairs sequenced (FPKM) value of each gene was calculated using cufflinks, and the read counts of each gene were obtained by htseq-count. Differentially expressed genes (DEGs) were identified using the DESeq. R package functions estimateSizeFactors and nbinomTest. *P* value < 0.05, fold Change > 2 and false discovery rate (FDR) < 0.05 were set as the threshold for significantly differential expression. Hierarchical cluster analysis of DEGs was performed to explore genes expression pattern.

### Gene ontology (GO) and KEGG pathway analysis

For miRNAs, GO enrichment and KEGG pathway enrichment analysis of different expressed miRNA-target-Gene were performed using R based on the hypergeometric distribution. For mRNAs, GO enrichment and KEGG pathway enrichment analysis of DEGs were performed using R based on the hypergeometric distribution [72].

### Identification of HMA gene family in wheat

The HMA gene family was identified following the method as described by Li et al. with some modifications [73]. First, to construct a local protein database, all the wheat (*T.aestivum L*.) protein sequences were downloaded from the Ensemble database (http://plants.ensembl.org/index.html). Then, the database were searched with 17 known HMA gene sequences collected from *A.thaliana* (8) and *O.sativa* (9) using the local BLASTP program with an e-value of le-5 and identity of 50% as the threshold. Moreover, a self-blast of these sequences was performed to remove redundant sequences. The physical localizations of all candidate HMA genes were checked and redundant sequences with the same chromosome location were rejected. To analyze whether there were domains belonging to the HMA gene family, the online tool InterProScan 5 (http://www.ebi.ac.uk/interpro/search/sequence search) [74] was used. Finally, to verify the existence of all the obtained sequences, BLASTN similarity search against the wheat ESTs deposited in the NCBI database were performed. The theorectical pI (isoelectric point) and Mw (molecular weight) of the putative HMA from T.aestivum L were calculated using compute pI/Mw tool online (http://web.expasy.org/compute_pi/), respectively.

### Gene structure construction, protein domain and motif analysis

Gene structure information was obtained from the Ensemble plants database (http://plants.ensembl.org/index.html). All full-length amino acid sequences of the TaHMAs were also used to identify conserved domain motifs by the Multiple Em for Motif Elicitation (MEME) tool [75]. The parameters were set as follows: maximum numbers of different motifs, 10; minimum motif width, 6; maximum motif width, 50.

### Phylogenetic analysis

The HMA protein sequences from *A.thaliana*, *O.sativa* and *T.aestivum L*. were performed for multiple alignments by CLUSTALW and the results of alignment were used to construct phylogenetic tree using the NJ method in MEGA (version 6.0) [76]. Bootstrap test method was emplyed and the replicate was set to 1000.

### The TaHMA gene expression analysis by RNA-seq data

To study the expression of TaHMA genes in different organs, the wheat expression database (http://wheat.pw.usda.gov/WheatExp/) was used to investigate the differential expression of TaHMAs. The FPKM (fragments per kilobase of transcript per million fragments mapped) value was calculated for each HMA gene, the log2 transformed FPKM value of the TaHMA genes were used for heat map generation. And *p*-value < 0.05 were taken as statistically significant threshold [77].

### Quantitative real time polymerase chain reaction (qRT-PCR) analysis

Eight miRNAs and six genes with different expression patterns and eight TaHMA genes were selected for validation by quantitative real-time RT-PCR (qRT-PCR). Total RNA was extracted from roots of L17 and H17 seedlings treated with 0 (CK) or 100 μM CdCl2 (Cd) for 24 h. For mRNA, first strand cDNA was synthesized using HiScript II Q RT SuperMix (Vazyme, R223–1). For miRNA, the first strand cDNA was synthesized using miScript II Reverse Transcription Kit (Qiagen, 218,161). The qRT-PCR was carried out using QuantiFast® SYBR® Green PCR kit (Qiagen, 204,054) according to the manufacturer’s instructions. The primers used in the qRT-PCR analyses were listed in (Additional file 4). GAPDH and 5S were used as internal genes for mRNAs and miRNAs, respectively. Three technical replicates were performed for each gene. The expression levels were calculated from the 2^-ΔΔCt^ value.

### Statistical analysis

All data were represented as mean ± SD and analyzed with SPSS20.0 software. For multiple group comparison, ANOVA with least significant difference was applied. *P* < 0.05 was considered statistically significant.

## Additional files

Additional file 1: Grain Cd concentration among wheat germplasms. (XLSX 16 kb)

Additional file 2: **Figure S1.** Grain Cd concentration between bread wheat and durum wheat. **Figure S2.** A Box plot of miRNA. B Correlation analysis of expression levels of miRNAs between samples and samples. **Figure S3.** A Box plot of mRNA. B Correlation analysis of expression levels of mRNAs between samples and samples. **Figure S4.** A The volcano plot illustrates the distribution of mRNA expression fold changes vs P values between L17Cd and L17CK. B The volcano plot illustrates the distribution of the data in mRNA profiles between H17Cd and HL17CK. Red, green and black points in the volcano plot represents significantly upregulated mRNAs, significantly downregulated mRNAs and not differential expressed mRNAs, respectively. **Figure S5.** GO enrichment analysis of differentially-expressed miRNA-targeted genes. The top 10 GOs enriched in targeted genes are given in response to miRNAs: (A) down-regulated and (B) up-regulated between L17Cd and L17CK; (C) down-regulated (D) up-regulated between H17Cd and H17CK. The y-axis represent the number of genes enriched. **Figure S6.** Venn diagrams of mRNA Gene Ontology (GO) enrichment results. Overlap of GO results of differentially expressed microRNAs (DEMs) between L17Cd and L17CK, H17Cd and H17CK. B Venn diagram for GO of DEGs between L17Cd and L17CK, H17Cd and H17CK. **Figure S7.** GO analysis for differentially-expressed mRNAs. The top 10 GOs enriched in differentially expressed mRNAs are given for the contrasts: (A) up-regulated and (B) down-regulated between L17Cd and L17CK; (C) up-regulated and (D) down-regulated between H17Cd and H17CK. The y-axis represent the gene number enriched in GOs. **Figure S8.** Ten wheat HMAs motifs were identified by MEME tools and indicated by different color. Motif location and combined p-value were represented. (ZIP 1392 kb)

Additional file 3: Functional annotation of transport genes. (XLSX 27 kb)

Additional file 4: Primers for genes. (XLSX 10 kb)

## Data Availability

The dataset and materials presented in the investigation is available by request from the corresponding author.

## Abbreviations

Cd: Cadmium
Chuanyu: Chuanyu wheat cultivars
CIMMYT: International Maize and Wheat Improvement Center
CMC: Chinese modern cultivars
DEGs: Differentially expressed genes
DEMs: Differentially expressed microRNAs
FDR: False discovery rate
FPKM: Fragments per kilobase of transcript per million fragments mapped
GO: Gene ontology
HMAs: Heavy metal ATPases
MEME: Multiple Em for Motif Elicitation
qRT-PCR: Quantitative real time quantitative-polymerase chain reaction
