# Supplementary figures and images for: The genome-wide impact of cadmium on microRNA and mRNA expression in contrasting Cd responsive wheat genotypes
Source: BMC Genomics. 2019 Jul 29;20:615. doi: 10.1186/s12864-019-5939-z (PMC6664702; doi:10.1186/s12864-019-5939-z)

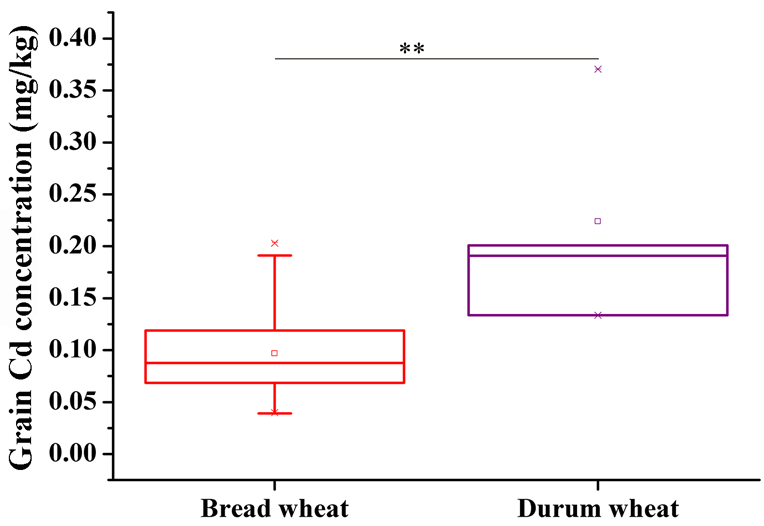

Supplement: Supplementary file 2 — Figure S1. Grain Cd concentration between bread wheat and durum wheat. Figure S2. A Box plot of miRNA. B Correlation analysis of expression levels of miRNAs between samples and samples. Figure S3. A Box plot of mRNA. B Correlation analysis of expression levels of mRNAs between samples and samples. Figure S4. A The volcano plot illustrates the distribution of mRNA expression fold changes vs P values between L17Cd and L17CK. B The volcano plot illustrates the distribution of the data in mRNA profiles between H17Cd and HL17CK. Red, green and black points in the volcano plot represents significantly upregulated mRNAs, significantly downregulated mRNAs and not differential expressed mRNAs, respectively. Figure S5. GO enrichment analysis of differentially-expressed miRNA-targeted genes. The top 10 GOs enriched in targeted genes are given in response to miRNAs: (A) down-regulated and (B) up-regulated between L17Cd and L17CK; (C) down-regulated (D) up-regulated between H17Cd and H17CK. The y-axis represent the number of genes enriched. Figure S6. Venn diagrams of mRNA Gene Ontology (GO) enrichment results. Overlap of GO results of differentially expressed microRNAs (DEMs) between L17Cd and L17CK, H17Cd and H17CK. B Venn diagram for GO of DEGs between L17Cd and L17CK, H17Cd and H17CK. Figure S7. GO analysis for differentially-expressed mRNAs. The top 10 GOs enriched in differentially expressed mRNAs are given for the contrasts: (A) up-regulated and (B) down-regulated between L17Cd and L17CK; (C) up-regulated and (D) down-regulated between H17Cd and H17CK. The y-axis represent the gene number enriched in GOs. Figure S8. Ten wheat HMAs motifs were identified by MEME tools and indicated by different color. Motif location and combined p-value were represented. (ZIP 1392 kb) [file 12864_2019_5939_MOESM2_ESM.zip › Additional file 2 Figure S1.tif]

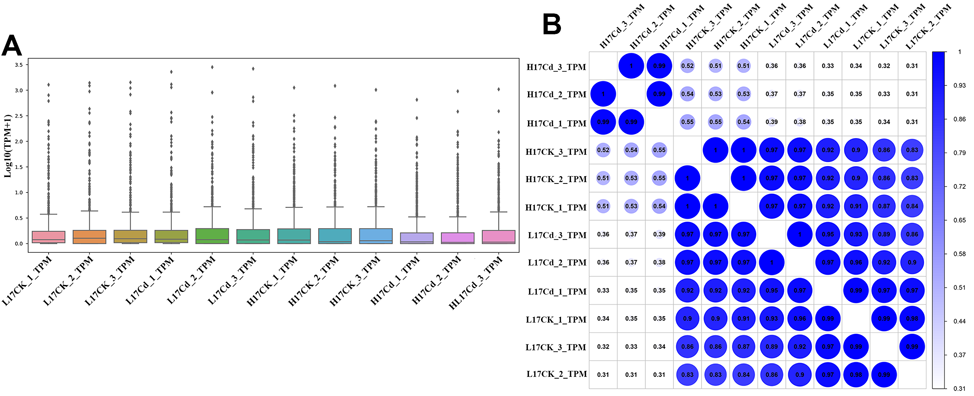

Supplement: Supplementary file 2 — Figure S1. Grain Cd concentration between bread wheat and durum wheat. Figure S2. A Box plot of miRNA. B Correlation analysis of expression levels of miRNAs between samples and samples. Figure S3. A Box plot of mRNA. B Correlation analysis of expression levels of mRNAs between samples and samples. Figure S4. A The volcano plot illustrates the distribution of mRNA expression fold changes vs P values between L17Cd and L17CK. B The volcano plot illustrates the distribution of the data in mRNA profiles between H17Cd and HL17CK. Red, green and black points in the volcano plot represents significantly upregulated mRNAs, significantly downregulated mRNAs and not differential expressed mRNAs, respectively. Figure S5. GO enrichment analysis of differentially-expressed miRNA-targeted genes. The top 10 GOs enriched in targeted genes are given in response to miRNAs: (A) down-regulated and (B) up-regulated between L17Cd and L17CK; (C) down-regulated (D) up-regulated between H17Cd and H17CK. The y-axis represent the number of genes enriched. Figure S6. Venn diagrams of mRNA Gene Ontology (GO) enrichment results. Overlap of GO results of differentially expressed microRNAs (DEMs) between L17Cd and L17CK, H17Cd and H17CK. B Venn diagram for GO of DEGs between L17Cd and L17CK, H17Cd and H17CK. Figure S7. GO analysis for differentially-expressed mRNAs. The top 10 GOs enriched in differentially expressed mRNAs are given for the contrasts: (A) up-regulated and (B) down-regulated between L17Cd and L17CK; (C) up-regulated and (D) down-regulated between H17Cd and H17CK. The y-axis represent the gene number enriched in GOs. Figure S8. Ten wheat HMAs motifs were identified by MEME tools and indicated by different color. Motif location and combined p-value were represented. (ZIP 1392 kb) [file 12864_2019_5939_MOESM2_ESM.zip › Additional file 2 Figure S2.tif]

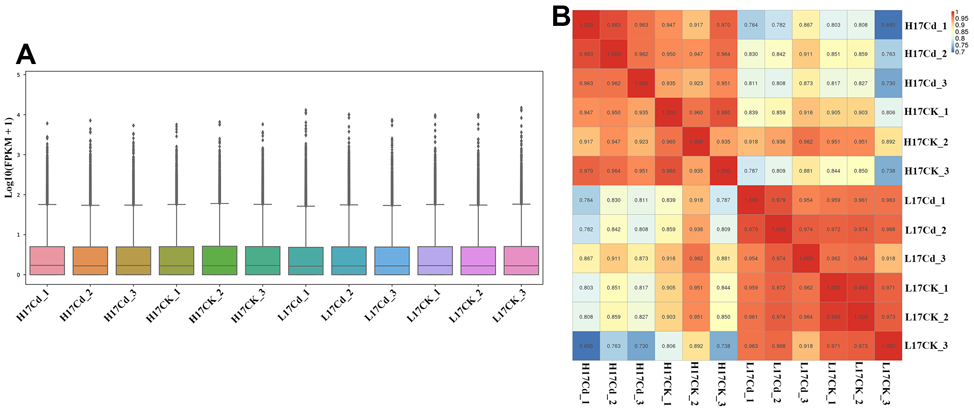

Supplement: Supplementary file 2 — Figure S1. Grain Cd concentration between bread wheat and durum wheat. Figure S2. A Box plot of miRNA. B Correlation analysis of expression levels of miRNAs between samples and samples. Figure S3. A Box plot of mRNA. B Correlation analysis of expression levels of mRNAs between samples and samples. Figure S4. A The volcano plot illustrates the distribution of mRNA expression fold changes vs P values between L17Cd and L17CK. B The volcano plot illustrates the distribution of the data in mRNA profiles between H17Cd and HL17CK. Red, green and black points in the volcano plot represents significantly upregulated mRNAs, significantly downregulated mRNAs and not differential expressed mRNAs, respectively. Figure S5. GO enrichment analysis of differentially-expressed miRNA-targeted genes. The top 10 GOs enriched in targeted genes are given in response to miRNAs: (A) down-regulated and (B) up-regulated between L17Cd and L17CK; (C) down-regulated (D) up-regulated between H17Cd and H17CK. The y-axis represent the number of genes enriched. Figure S6. Venn diagrams of mRNA Gene Ontology (GO) enrichment results. Overlap of GO results of differentially expressed microRNAs (DEMs) between L17Cd and L17CK, H17Cd and H17CK. B Venn diagram for GO of DEGs between L17Cd and L17CK, H17Cd and H17CK. Figure S7. GO analysis for differentially-expressed mRNAs. The top 10 GOs enriched in differentially expressed mRNAs are given for the contrasts: (A) up-regulated and (B) down-regulated between L17Cd and L17CK; (C) up-regulated and (D) down-regulated between H17Cd and H17CK. The y-axis represent the gene number enriched in GOs. Figure S8. Ten wheat HMAs motifs were identified by MEME tools and indicated by different color. Motif location and combined p-value were represented. (ZIP 1392 kb) [file 12864_2019_5939_MOESM2_ESM.zip › Additional file 2 Figure S3.tif]

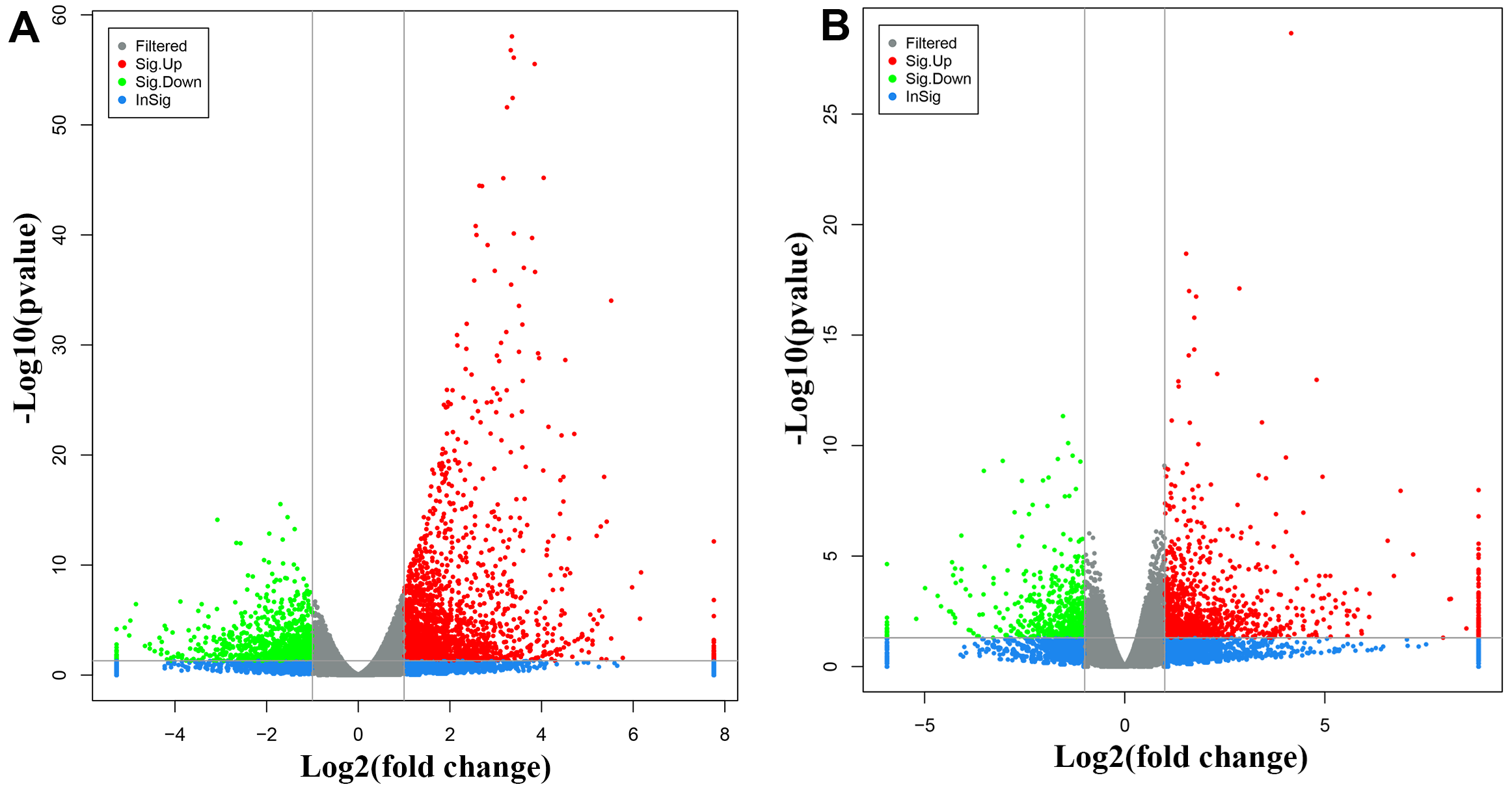

Supplement: Supplementary file 2 — Figure S1. Grain Cd concentration between bread wheat and durum wheat. Figure S2. A Box plot of miRNA. B Correlation analysis of expression levels of miRNAs between samples and samples. Figure S3. A Box plot of mRNA. B Correlation analysis of expression levels of mRNAs between samples and samples. Figure S4. A The volcano plot illustrates the distribution of mRNA expression fold changes vs P values between L17Cd and L17CK. B The volcano plot illustrates the distribution of the data in mRNA profiles between H17Cd and HL17CK. Red, green and black points in the volcano plot represents significantly upregulated mRNAs, significantly downregulated mRNAs and not differential expressed mRNAs, respectively. Figure S5. GO enrichment analysis of differentially-expressed miRNA-targeted genes. The top 10 GOs enriched in targeted genes are given in response to miRNAs: (A) down-regulated and (B) up-regulated between L17Cd and L17CK; (C) down-regulated (D) up-regulated between H17Cd and H17CK. The y-axis represent the number of genes enriched. Figure S6. Venn diagrams of mRNA Gene Ontology (GO) enrichment results. Overlap of GO results of differentially expressed microRNAs (DEMs) between L17Cd and L17CK, H17Cd and H17CK. B Venn diagram for GO of DEGs between L17Cd and L17CK, H17Cd and H17CK. Figure S7. GO analysis for differentially-expressed mRNAs. The top 10 GOs enriched in differentially expressed mRNAs are given for the contrasts: (A) up-regulated and (B) down-regulated between L17Cd and L17CK; (C) up-regulated and (D) down-regulated between H17Cd and H17CK. The y-axis represent the gene number enriched in GOs. Figure S8. Ten wheat HMAs motifs were identified by MEME tools and indicated by different color. Motif location and combined p-value were represented. (ZIP 1392 kb) [file 12864_2019_5939_MOESM2_ESM.zip › Additional file 2 Figure S4.tif]

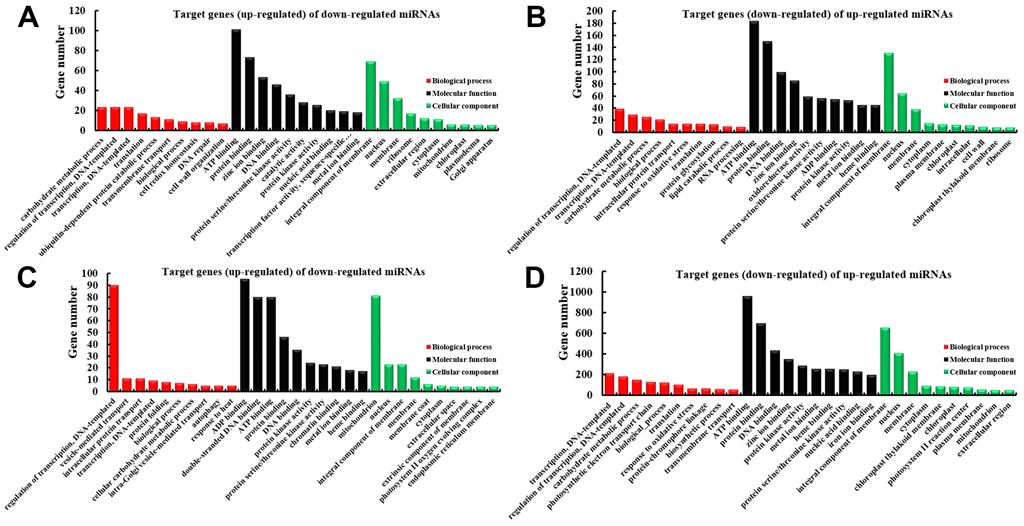

Supplement: Supplementary file 2 — Figure S1. Grain Cd concentration between bread wheat and durum wheat. Figure S2. A Box plot of miRNA. B Correlation analysis of expression levels of miRNAs between samples and samples. Figure S3. A Box plot of mRNA. B Correlation analysis of expression levels of mRNAs between samples and samples. Figure S4. A The volcano plot illustrates the distribution of mRNA expression fold changes vs P values between L17Cd and L17CK. B The volcano plot illustrates the distribution of the data in mRNA profiles between H17Cd and HL17CK. Red, green and black points in the volcano plot represents significantly upregulated mRNAs, significantly downregulated mRNAs and not differential expressed mRNAs, respectively. Figure S5. GO enrichment analysis of differentially-expressed miRNA-targeted genes. The top 10 GOs enriched in targeted genes are given in response to miRNAs: (A) down-regulated and (B) up-regulated between L17Cd and L17CK; (C) down-regulated (D) up-regulated between H17Cd and H17CK. The y-axis represent the number of genes enriched. Figure S6. Venn diagrams of mRNA Gene Ontology (GO) enrichment results. Overlap of GO results of differentially expressed microRNAs (DEMs) between L17Cd and L17CK, H17Cd and H17CK. B Venn diagram for GO of DEGs between L17Cd and L17CK, H17Cd and H17CK. Figure S7. GO analysis for differentially-expressed mRNAs. The top 10 GOs enriched in differentially expressed mRNAs are given for the contrasts: (A) up-regulated and (B) down-regulated between L17Cd and L17CK; (C) up-regulated and (D) down-regulated between H17Cd and H17CK. The y-axis represent the gene number enriched in GOs. Figure S8. Ten wheat HMAs motifs were identified by MEME tools and indicated by different color. Motif location and combined p-value were represented. (ZIP 1392 kb) [file 12864_2019_5939_MOESM2_ESM.zip › Additional file 2 Figure S5.tif]

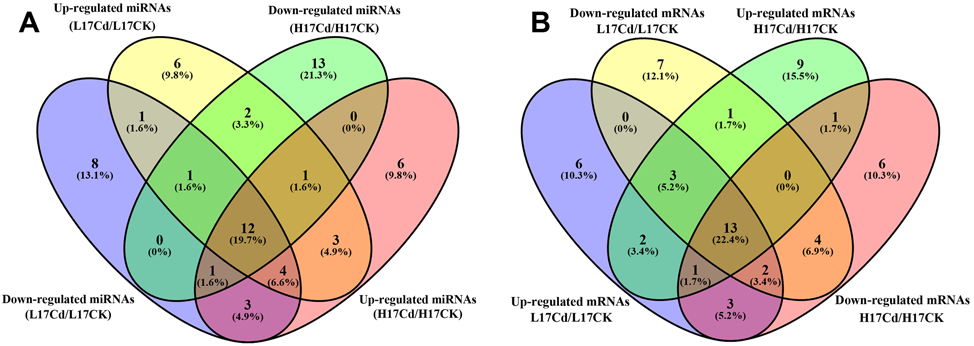

Supplement: Supplementary file 2 — Figure S1. Grain Cd concentration between bread wheat and durum wheat. Figure S2. A Box plot of miRNA. B Correlation analysis of expression levels of miRNAs between samples and samples. Figure S3. A Box plot of mRNA. B Correlation analysis of expression levels of mRNAs between samples and samples. Figure S4. A The volcano plot illustrates the distribution of mRNA expression fold changes vs P values between L17Cd and L17CK. B The volcano plot illustrates the distribution of the data in mRNA profiles between H17Cd and HL17CK. Red, green and black points in the volcano plot represents significantly upregulated mRNAs, significantly downregulated mRNAs and not differential expressed mRNAs, respectively. Figure S5. GO enrichment analysis of differentially-expressed miRNA-targeted genes. The top 10 GOs enriched in targeted genes are given in response to miRNAs: (A) down-regulated and (B) up-regulated between L17Cd and L17CK; (C) down-regulated (D) up-regulated between H17Cd and H17CK. The y-axis represent the number of genes enriched. Figure S6. Venn diagrams of mRNA Gene Ontology (GO) enrichment results. Overlap of GO results of differentially expressed microRNAs (DEMs) between L17Cd and L17CK, H17Cd and H17CK. B Venn diagram for GO of DEGs between L17Cd and L17CK, H17Cd and H17CK. Figure S7. GO analysis for differentially-expressed mRNAs. The top 10 GOs enriched in differentially expressed mRNAs are given for the contrasts: (A) up-regulated and (B) down-regulated between L17Cd and L17CK; (C) up-regulated and (D) down-regulated between H17Cd and H17CK. The y-axis represent the gene number enriched in GOs. Figure S8. Ten wheat HMAs motifs were identified by MEME tools and indicated by different color. Motif location and combined p-value were represented. (ZIP 1392 kb) [file 12864_2019_5939_MOESM2_ESM.zip › Additional file 2 Figure S6.tif]

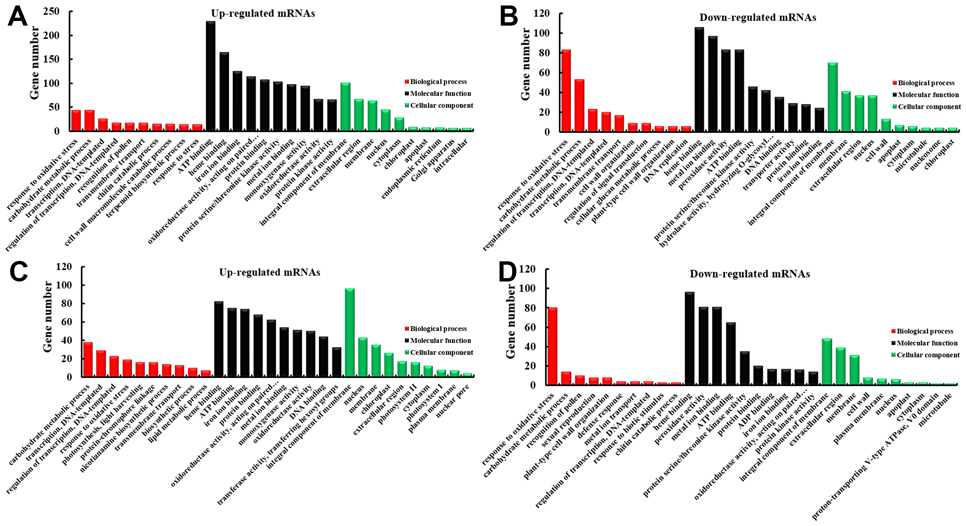

Supplement: Supplementary file 2 — Figure S1. Grain Cd concentration between bread wheat and durum wheat. Figure S2. A Box plot of miRNA. B Correlation analysis of expression levels of miRNAs between samples and samples. Figure S3. A Box plot of mRNA. B Correlation analysis of expression levels of mRNAs between samples and samples. Figure S4. A The volcano plot illustrates the distribution of mRNA expression fold changes vs P values between L17Cd and L17CK. B The volcano plot illustrates the distribution of the data in mRNA profiles between H17Cd and HL17CK. Red, green and black points in the volcano plot represents significantly upregulated mRNAs, significantly downregulated mRNAs and not differential expressed mRNAs, respectively. Figure S5. GO enrichment analysis of differentially-expressed miRNA-targeted genes. The top 10 GOs enriched in targeted genes are given in response to miRNAs: (A) down-regulated and (B) up-regulated between L17Cd and L17CK; (C) down-regulated (D) up-regulated between H17Cd and H17CK. The y-axis represent the number of genes enriched. Figure S6. Venn diagrams of mRNA Gene Ontology (GO) enrichment results. Overlap of GO results of differentially expressed microRNAs (DEMs) between L17Cd and L17CK, H17Cd and H17CK. B Venn diagram for GO of DEGs between L17Cd and L17CK, H17Cd and H17CK. Figure S7. GO analysis for differentially-expressed mRNAs. The top 10 GOs enriched in differentially expressed mRNAs are given for the contrasts: (A) up-regulated and (B) down-regulated between L17Cd and L17CK; (C) up-regulated and (D) down-regulated between H17Cd and H17CK. The y-axis represent the gene number enriched in GOs. Figure S8. Ten wheat HMAs motifs were identified by MEME tools and indicated by different color. Motif location and combined p-value were represented. (ZIP 1392 kb) [file 12864_2019_5939_MOESM2_ESM.zip › Additional file 2 Figure S7.tif]

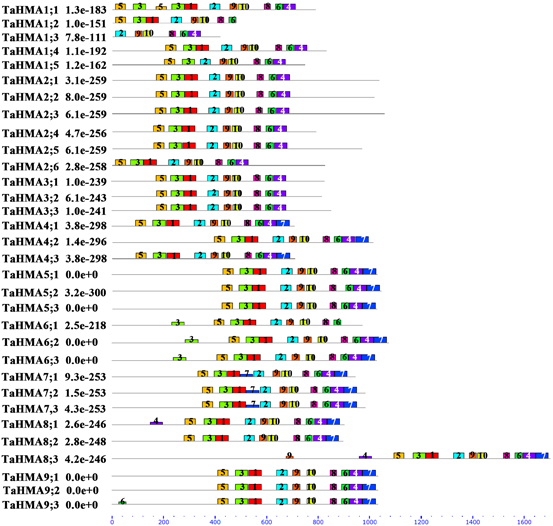

Supplement: Supplementary file 2 — Figure S1. Grain Cd concentration between bread wheat and durum wheat. Figure S2. A Box plot of miRNA. B Correlation analysis of expression levels of miRNAs between samples and samples. Figure S3. A Box plot of mRNA. B Correlation analysis of expression levels of mRNAs between samples and samples. Figure S4. A The volcano plot illustrates the distribution of mRNA expression fold changes vs P values between L17Cd and L17CK. B The volcano plot illustrates the distribution of the data in mRNA profiles between H17Cd and HL17CK. Red, green and black points in the volcano plot represents significantly upregulated mRNAs, significantly downregulated mRNAs and not differential expressed mRNAs, respectively. Figure S5. GO enrichment analysis of differentially-expressed miRNA-targeted genes. The top 10 GOs enriched in targeted genes are given in response to miRNAs: (A) down-regulated and (B) up-regulated between L17Cd and L17CK; (C) down-regulated (D) up-regulated between H17Cd and H17CK. The y-axis represent the number of genes enriched. Figure S6. Venn diagrams of mRNA Gene Ontology (GO) enrichment results. Overlap of GO results of differentially expressed microRNAs (DEMs) between L17Cd and L17CK, H17Cd and H17CK. B Venn diagram for GO of DEGs between L17Cd and L17CK, H17Cd and H17CK. Figure S7. GO analysis for differentially-expressed mRNAs. The top 10 GOs enriched in differentially expressed mRNAs are given for the contrasts: (A) up-regulated and (B) down-regulated between L17Cd and L17CK; (C) up-regulated and (D) down-regulated between H17Cd and H17CK. The y-axis represent the gene number enriched in GOs. Figure S8. Ten wheat HMAs motifs were identified by MEME tools and indicated by different color. Motif location and combined p-value were represented. (ZIP 1392 kb) [file 12864_2019_5939_MOESM2_ESM.zip › Additional file 2 Figure S8.tif]
